# Elevated Inflammation Associated with Markers of Neutrophil Function and Gastrointestinal Disruption in Pilot Study of *Plasmodium fragile* Co-Infection of ART-Treated SIVmac239+ Rhesus Macaques

**DOI:** 10.3390/v16071036

**Published:** 2024-06-27

**Authors:** Sydney M. Nemphos, Hannah C. Green, James E. Prusak, Sallie L. Fell, Kelly Goff, Megan Varnado, Kaitlin Didier, Natalie Guy, Matilda J. Moström, Coty Tatum, Chad Massey, Mary B. Barnes, Lori A. Rowe, Carolina Allers, Robert V. Blair, Monica E. Embers, Nicholas J. Maness, Preston A. Marx, Brooke Grasperge, Amitinder Kaur, Kristina De Paris, Jeffrey G. Shaffer, Tiffany Hensley-McBain, Berlin Londono-Renteria, Jennifer A. Manuzak

**Affiliations:** 1 Division of Immunology, Tulane National Primate Research Center, Covington, LA 70433, USA; 2 Division of Microbiology, Tulane National Primate Research Center, Covington, LA 70433, USA; 3 Division of Comparative Pathology, Tulane National Primate Research Center, Covington, LA 70433, USA; 4 Department of Microbiology and Immunology, Tulane University School of Medicine, New Orleans, LA 70112, USA; 5 Department of Tropical Medicine and Infectious Disease, Tulane University School of Public Health and Tropical Medicine, New Orleans, LA 70112, USA; blondono@tulane.edu; 6 Division of Veterinary Medicine, Tulane National Primate Research Center, Covington, LA 70433, USA; 7 Department of Microbiology and Immunology, University of North Carolina School of Medicine, Chapel Hill, NC 27559, USA; 8 Department of Biostatistics and Data Science, Tulane University School of Public Health and Tropical Medicine, New Orleans, LA 70112, USA; 9 McLaughlin Research Institute for Biomedical Sciences, Great Falls, MT 59405, USA

**Keywords:** nonhuman primate, malaria, *Plasmodium fragile*, simian immunodeficiency virus, neutrophils, co-infection, immunology

## Abstract

Human immunodeficiency virus (HIV) and malaria, caused by infection with *Plasmodium* spp., are endemic in similar geographical locations. As a result, there is high potential for HIV/*Plasmodium* co-infection, which increases the pathology of both diseases. However, the immunological mechanisms underlying the exacerbated disease pathology observed in co-infected individuals are poorly understood. Moreover, there is limited data available on the impact of *Plasmodium* co-infection on antiretroviral (ART)-treated HIV infection. Here, we used the rhesus macaque (RM) model to conduct a pilot study to establish a model of *Plasmodium fragile* co-infection during ART-treated simian immunodeficiency virus (SIV) infection, and to begin to characterize the immunopathogenic effect of co-infection in the context of ART. We observed that *P. fragile* co-infection resulted in parasitemia and anemia, as well as persistently detectable viral loads (VLs) and decreased absolute CD4+ T-cell counts despite daily ART treatment. Notably, *P. fragile* co-infection was associated with increased levels of inflammatory cytokines, including monocyte chemoattractant protein 1 (MCP-1). *P. fragile* co-infection was also associated with increased levels of neutrophil elastase, a plasma marker of neutrophil extracellular trap (NET) formation, but significant decreases in markers of neutrophil degranulation, potentially indicating a shift in the neutrophil functionality during co-infection. Finally, we characterized the levels of plasma markers of gastrointestinal (GI) barrier permeability and microbial translocation and observed significant correlations between indicators of GI dysfunction, clinical markers of SIV and *Plasmodium* infection, and neutrophil frequency and function. Taken together, these pilot data verify the utility of using the RM model to examine ART-treated SIV/*P. fragile* co-infection, and indicate that neutrophil-driven inflammation and GI dysfunction may underlie heightened SIV/*P. fragile* co-infection pathogenesis.

## 1. Introduction

Human immunodeficiency virus (HIV) and malaria, caused by *Plasmodium* parasites, are two of the world’s most devastating infections. In 2022, over 39 million people were living with HIV (PWH) [1], and there were over 247 million cases of malaria [2]. Despite effective tools and treatments, challenges in the prevention and eradication of both infections remain. Indeed, consistent use of antiretrovirals (ART) allows for sustained viral suppression, improving the health and quality of life of PWH [3]. However, ART does not eliminate the viral reservoir, and plasma viremia rapidly rebounds post-treatment-interruption [4,5]. Similarly, antimalarial drugs can prevent and cure *Plasmodium* infection, but the emergence of drug resistance undermines control efforts and contributes to increased morbidity and mortality [6,7]. For both, the complexities of the infectious agents, combined with an incomplete knowledge of the immunological mechanisms underlying pathogenesis, hampers the identification of immune correlates of protection and development of fully efficacious prophylactic vaccines.

HIV and malaria endemicity are geographically overlapped, creating high potential for co-infection. A meta-analysis of studies conducted between 1991 and 2018 found that the co-incidence of HIV and severe malaria, defined as the presence of peripheral parasitemia in combination with manifestations such as severe anemia or multi-organ failure, was 43% [8]. Prior work has demonstrated reciprocal antagonistic effects that result in the increased transmission of both HIV and malaria. For example, *Plasmodium* increases HIV viral loads (VLs) both in vitro and in ART-naïve PWH [9,10,11]. High VLs correlate with increased HIV transmission, suggesting that malaria co-infection in PWH could enhance HIV transmission risk [12,13,14]. Similarly, clinical malaria prevalence, malaria infection severity, and malaria-associated mortality rates are increased in ART-naïve PWH [15,16,17,18]. Additionally, previous in vitro and ex vivo studies have indicated that co-infection with both HIV and *Plasmodium* exacerbates disease pathogenesis. For example, in vitro infection of monocyte-derived macrophages with a laboratory strain of HIV-1 resulted in the inhibition of phagocytic capability and cytokine production in response to stimulation with opsonized trophozoites from a laboratory-adapted strain of *P. falciparum* [19]. Moreover, HIV-infected children in Malawi with cerebral malaria infection had higher rates of and more rapid progression to mortality, greater parasite loads in the brain and spleen, and a greater accumulation of monocytes and platelets in the brain, as compared to children without HIV [20,21]. Notably, uncontrolled inflammation underlies disease pathogenesis in separate HIV and malaria infection [22,23]. In PWH, increased inflammation is associated with viral persistence, disruptions in intestinal homeostasis, and an increased risk of co-infection with other pathogens [24]. Likewise, a pro-inflammatory environment is associated with severe malaria and increased malaria-associated mortality [23,25]. Importantly, the impact of *Plasmodium* co-infection on ART efficacy in PWH, and the link between inflammatory responses and disease pathology during co-infection, have not yet been fully defined [26].

Neutrophils are granulocytes that constitute up to 70% of all circulating leukocytes [27] and aid in host defense through (1) exocytosis of anti-microbial molecule-containing granules; (2) phagocytosis and destruction of microbes in phagosomes; and (3) the formation of neutrophil extracellular traps (NETs), DNA decorated with granule contents that aid pathogen clearance [28,29,30,31]. Conversely, dysregulated neutrophil activation causes uncontrolled inflammation and collateral host tissue damage [32]. For example, although NET formation is associated with protection against HIV infection and impaired replication in vitro [33], increased neutrophil activation has been linked with adverse clinical outcomes in PWH [34,35,36], and ART-treated PWH exhibited impaired neutrophil phagocytosis and increased neutrophil apoptosis, as compared to uninfected controls [37,38]. Additionally, increased neutrophil infiltration into the gastrointestinal (GI) mucosa in ART-treated PWH and macaques with chronic simian immunodeficiency virus (SIV) is associated with the loss of GI epithelial barrier integrity and elevated microbial translocation [39,40,41], both of which are associated with chronic inflammation, morbidity, and mortality in PWH [42,43,44]. Likewise, neutrophil phagocytosis aids in *Plasmodium* clearance [45,46,47] and NET formation in children and is associated with parasite killing [48]. However, NET formation is also associated with increased inflammation and severe malaria [49,50,51], and excessive neutrophil degranulation contributed to risk for severe malaria [48,52,53]. Importantly, the role of neutrophils in disease pathogenesis during HIV/*Plasmodium* co-infection has not been established.

SIV infection induces pathologies similar to progressive HIV, including high peak and chronic plasma VLs and CD4+ T-cell depletion [54]. Additionally, like *P. falciparum*, which causes most human malaria cases worldwide [2], *P. fragile* is capable of endothelial adherence, tissue sequestration, and antigenic variation in rhesus macaques (RMs) [55]. SIV/*P. fragile* co-infection in RMs mimics HIV/*P. falciparum* co-infection in humans, including increased SIV VLs and innate immune dysfunction in ART-naïve SIV/*P. fragile*-co-infected versus singly infected RMs [56,57]. However, SIV/*P. fragile* co-infection has not been characterized in the context of ART. In this pilot study, we sought to verify the utility of using the RM model to examine ART-treated SIV/*P. fragile* co-infection, and to begin defining the immunopathogenic effect of SIV/*P. fragile* co-infection in the context of ART. We hypothesized that *P. fragile* co-infection would result in exacerbated SIV pathology that associated with neutrophil dysfunction despite ART. To test this hypothesis, we infected four adult RMs with SIVmac239, followed by ART initiation and *P. fragile* co-infection, and we longitudinally monitored clinical and immune markers.

## 2. Methods and Materials

### 2.1. Study Animals and Approval

Four adult (aged 6–12 years), male, Indian-origin RMs were housed and cared for at the Tulane National Primate Research Center (TNPRC) under an Institutional Animal Care and Use Committee (IACUC; Office of Laboratory Animal Welfare Assurance Number A4499-01)-approved protocol (P0477-3564). Animal housing, care, and procedures were performed at Association for Assessment and Accreditation of Laboratory Animal Care-accredited facilities (AAALAC Number 000594), compliant with United States Department of Agriculture regulations, including the Animal Welfare Act (9 CFR) and the Animal Care Policy Manual, with guidelines established by the National Research Council in the Guide for the Care and Use of Laboratory Animals and the Weatherall Report. All animals were naïve for both SIV and *Plasmodium* prior to study assignment. In addition, animals were negative for MHC class I alleles associated with SIV control, including Mamu-A*01, Mamu-B*08, and Mamu-B*17 [58,59,60]. Animals were singly housed indoors under climate-controlled conditions and a 12 h light/12 h dark cycle, and were monitored daily to ensure welfare. Abnormalities were recorded and reported to a veterinarian. Water was available ad libitum and animals were fed commercial monkey chow (Purina LabDiet; PMI Nutrition International, Richmond, IN), supplemented with fruits, vegetables, and foraging treats as a part of the TNPRC environmental enrichment program. At week 2 post-SIV-infection (p.i.), one animal (LN07) received a topical antibiotic for a surface wound. At weeks 10 and 13 p.i., all RMs received Kefzol (6.25 mg/kg) during surgical procedures. At week 14 p.i. one animal (LE96) received a blood transfusion. At weeks 14 (LC40) and 16 (LC40, LE96) p.i., two animals received a dose of the antibiotic Excede (200 mg/mL). Procedures were performed under the direction of TNPRC veterinarians. Anesthesia was used in accordance with the TNPRC policy and Weatherall Report. Euthanasia at the study endpoint was performed using methods consistent with the recommendations of the American Veterinary Medical Association and per the recommendations of the IACUC.

### 2.2. SIV Inoculation, Monitoring, and ART Treatment

RMs were intravenously inoculated with 50 TCID50 SIVmac239 [61]. Plasma VLs were monitored via RT-qPCR (lower limit of detection = 83 copies/mL) [62]. Starting at week 8 p.i. and continuing until endpoint, RMs received daily ART, administered subcutaneously, consisting of tenofovir disoproxil fumarate (TDF) (5.1 mg/kg), emtricitabine (FTC) (30 mg/kg; both from Gilead, Foster City, CA, USA), and dolutegravir (DTG) (2.5 mg/kg; ViiV Healthcare, London, England, UK), formulated in Kleptose (15% in 0.1 N NaOH, Roquette, Lestrem, France), a formulation selected for its effectiveness in suppressing SIV replication in RMs [63]. ART was started at week 8 p.i. in order to allow time for RMs to reach viral setpoint and establish late acute/early chronic SIVmac239 infection, as well as to model time to ART initiation in HIV/malaria co-endemic areas [64,65,66,67,68].

### 2.3. P. fragile Inoculation, Monitoring, and Antimalarial Treatment

RMs were intravenously inoculated with 20 × 10^6^ *P. fragile*-infected erythrocytes (Sri Lanka strain) [69,70,71]. Briefly, cryopreserved erythrocytes were thawed and resuspended in 12% NaCl (Thermo Fisher Scientific, Waltham, MA, USA) for 5 min at room temperature (RT). Next, 1.6% NaCl was added dropwise, followed by centrifugation for 10 min at RT and 1400 revolutions per minute (RPMs). The pellet was resuspended in 0.9% NaCl and 2% dextrose (Thermo Fisher Scientific), centrifuged for 10 min at RT and 1400 RPM, and resuspended in 0.9% of NaCl for inoculation. Anemia was monitored via hematocrit (HCT), calculated as the ratio of erythrocytes to the total blood volume. Parasitemia was monitored via Giemsa staining of thin blood smears collected from sedated animals via venipuncture or tail sticks from non-sedated animals using positive reinforcement, three days a week starting in week 12, delineated as week A, B, or C. Smears were fixed in methanol for 5 min, followed by staining in 5% Giemsa solution (pH = 7.2) for 45–60 min and then washed in distilled water. Parasitemia was calculated as the average number of parasitized erythrocytes among all erythrocytes in 10 randomized fields of view. Post-week 14A p.i., RMs received antimalarial drugs via oral gavage consisting of one administration of quinine sulfate (150 mg; Archway Apothecary, Covington, LA, USA; NDC: 51927-1588-00), followed by four daily administrations of chloroquine (20 mg/kg; Health Warehouse, Florence, KY, USA; NDC: 64980-0177-50).

### 2.4. Sample Collection and Processing

EDTA and serum gel vacutainer tubes (Starstedt, Newton, NC, USA) were used to collect peripheral blood. Complete blood counts (CBCs) were performed using EDTA blood on a Sysmex XN-1000v (Sysmex, Kobe, Hyogo, Japan). Blood chemistry was performed using fresh serum, and C-reactive protein (CRP) levels were quantified using frozen serum on a Beckman AU480 (Beckman, Brea, CA, USA). For experimental procedures, EDTA blood was centrifuged for 10 min at 2000 RPM and RT to isolate plasma, which was stored at −80 °C. After plasma removal, whole blood was reconstituted with PBS. Aliquots of 250 µL of blood–PBS were set aside for flow cytometric staining. Peripheral blood mononuclear cells (PBMCs) were isolated from the remaining blood via density-gradient centrifugation using Ficoll-Paque Plus (Sigma-Aldrich, St. Louis, MO, USA) and Accuspin tubes (Sigma-Aldrich). PBMCs were cryopreserved in freezing media (5 mls dimethyl sulfoxide (DMSO) [Sigma-Aldrich] + 45 mls heat-inactivated fetal bovine serum [Thermo Fisher Scientific]) and stored in liquid nitrogen.

### 2.5. Flow Cytometry

Multicolor flow cytometry was performed on whole blood using RM cross-reactive monoclonal antibodies. Samples were first stained with a Live/Dead Fixable Aqua dead-cell stain (Thermo Fisher Scientific) and were then treated with Fc block (BD Biosciences, Franklin Lakes, NJ, USA). Extracellular staining was performed using predetermined fluorochrome-conjugated antibody concentrations (Supplemental Table S1), followed by red blood cell lysis using a 1x FACS lysing solution (BD Biosciences). Cells were fixed, permeabilized (CytoFix/Perm Kit, BD Biosciences), and then intracellularly stained (Supplemental Table S1 and Supplemental Figure S1).

Phagocytosis was evaluated using *E. coli* bioparticles conjugated to a dye that fluoresces in acidic environments (pHrodo Red *E. coli* Bioparticles Phagocytosis Kit for Flow Cytometry; Thermo Fisher Scientific). Briefly, pHrodo bioparticles were incubated with plasma from healthy RMs (1:3 plasma:pHrodo ratio) for 30 min to allow for bioparticle opsonization. Opsonized pHrodo bioparticles were added to 250 µL blood-PBS aliquots for 2 h at 37 °C, followed by surface staining (Supplemental Figure S2).

All samples were fixed with 1% paraformaldehyde and held at 4 °C until acquisition on a BD LSRFortessa using FACSDiva software (v9.0). Single-color controls were acquired in every experiment as compensation. Analysis was performed using FlowJo (v10). In all analyses, individual cell subsets with less than 100 parental gate events were not included in the downstream analysis due to the inability to ensure adequate fluorescence separation.

CD4+ T lymphocyte kinetics were monitored by flow cytometric evaluation of absolute counts. Briefly, 50 μL of whole blood was surface-stained (Supplemental Table S2) and incubated for 20 min at RT in the dark. Red blood cells were lysed with a 1× BD FACS Lysing Solution for 30–45 min. The sample was mixed and volumetrically analyzed on a Miltenyi MACSQuant 16 (Miltenyi, Bergisch Gladbach, Germany).

### 2.6. Detection of Plasma Markers of Neutrophil Function, GI Mucosal Barrier Integrity, and Microbial Translocation

Commercially available enzyme-linked immunosorbent assay (ELISA) kits were used to quantify the plasma levels of neutrophil granule components, including myeloperoxidase (MPO) (Abcam, Cambridge, UK); cathepsin G (MyBiosource, San Diego, CA, USA); proteinase 3 (PR3) (MyBiosource); biomarkers of NET formation, including citrullinated histone 3 (CitH3) (Cayman Chemicals, Ann Arbor, MI, USA) and neutrophil elastase (NE) (LSBio, Lynwood, WA, USA); markers of GI barrier permeability, including Zonulin-1 (MyBiosource) and intestinal fatty acid-binding protein (IFAB-P) (Novus Biologicals, Centennial, CO, USA); and surrogate markers of microbial translocation, including lipopolysaccharide (LPS)-binding protein (LBP) (Novus Biologicals) and soluble CD14 (sCD14) ELISA (ThermoFisher), as per the manufacturers’ recommended protocols.

### 2.7. Detection of Plasma Markers of Systemic Inflammation

A BioLegend (San Diego, CA, USA) LEGENDplex™ NHP Inflammation Panel (13-plex) with a V-bottom plate was used to quantify plasma levels of IL-6, IL-10, CXCL10 (IP-10), IL-1β, IL-12p40, IL-17A, IFN-β, IL-23, TNF-α, IFN-γ, GM-CSF, CXCL8 (IL-8), and CCL2 (MCP-1). Plasma samples were run in duplicate at a 1:4 dilution, as per the manufacturer’s recommendations. Samples were acquired on a Miltenyi MACSQuant 16 in a 96-well-plate format. Data were analyzed with BioLegend LEGENDplex™ online analysis software (https://www.biolegend.com/de-de/immunoassays/legendplex) against a standard curve.

### 2.8. Data and Statistical Analysis

The absolute number of neutrophils/microliter of blood was used to calculate the number of neutrophils positive for pHrodo bioparticles to characterize phagocytosis (phagocytic score, or the number of neutrophils capable of phagocytosis) [72]. The phagocytic index, representing phagocytic proficiency, was calculated by multiplying the phagocytic score by the Mean Fluorescence Intensity of pHrodo-positive neutrophils [73].

Statistical significance was first calculated by using a mixed-effects analysis with the Geisser–Greenhouse correction. Individual comparisons were made between all measured timepoints with corrections for multiple comparisons performed using Tukey’s multiple comparison tests, with individual variances computed for each comparison. In all figures, multiplicity-adjusted significant *p* values are shown above horizontal black bars. To further calculate statistical significance, we next used a mixed-effects analysis with the Geisser–Greenhouse correction to compare all post-SIV and post-co-infection timepoints to baseline. These individual post-infection comparisons to baseline were conducted with corrections for multiple comparisons performed using a Dunnett’s multiple-comparison test, with individual variances computed for each comparison. Data on post-infection timepoints compared to the baseline are depicted in Supplemental Tables S3–S10.

Multivariate analysis of variance (MANOVA) approaches were applied to identify potential relationships between various neutrophil measures, clinical signs of both SIV and *P. fragile* infection, peripheral markers of inflammation, and peripheral markers of GI dysfunction. The MANOVA approach allowed us to account for temporal dependence, as previously described [74]. MANOVA was used to model 14 parameters (VL; anemia; absolute CD4+ count; peripheral neutrophils; plasma zonulin; sCD14; I-FABP; LBP; NE; cathepsin G; CitH3; IP-10; MCP-1; and CRP) against animal number. Partial correlation coefficients were generated based on the MANOVA error terms adjusted for animal effects. Statistical analyses were performed using GraphPad Prism (Version 10; GraphPad Software, San Diego, CA, USA) and the Statistical Analysis System (SAS) (Version 9.4; Cary, NC, USA). All reported *p* values were multiplicity-adjusted, and values of <0.05 were considered significant. The JoinPoint Regression Program (NIH, V5.0.2, Bethesda, MD, USA) was utilized to assess longitudinal trends in VLs.

## 3. Results

### 3.1. Experimental Design

**Figure 1:**
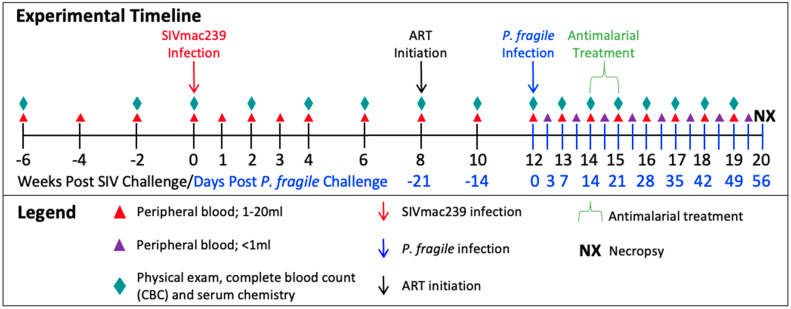
Experimental timeline depicting sample collection from adult, male rhesus macaques (RMs) (n = 4). RMs were inoculated with SIVmac239, TCID50 = 50, intravenously (i.v.) at week 0. Daily antiretroviral treatment (ART) was given subcutaneously, beginning at week 8, and continued until the end of the study (TDF/FTC/DTG; 5.1/30/2.5 mg/kg). RMs were inoculated with *Plasmodium fragile,* 20 × 10^6^-infected erythrocytes, via i.v. Antimalaria treatment occurred over one week, at week 14, and consisted of one oral gavage of quinine sulfate (150 mg) followed by four daily oral gavages of chloroquine (20 mg/kg).

Following baseline (BL) sampling, RMs (n = 4) were intravenously inoculated with SIVmac239 (Figure 1). At week 8 post-SIV infection (p.i.), RMs initiated ART, which continued until euthanasia at week 20 p.i. For simplicity, all timepoints in our analyses are reported as post-SIV infection. At week 12 p.i., RMs were intravenously inoculated with *P. fragile*. At week 14 p.i., RMs surpassed our treatment threshold of 0.5% parasitemia and received antimalarial drugs via oral gavage. Physical exams, peripheral-blood collections, CBCs, and serum chemistries were conducted throughout the study.

### 3.2. P. fragile Co-Infection of ART-Treated SIV+ RMs Results in Clinical Signs of Malaria

**Figure 2:**
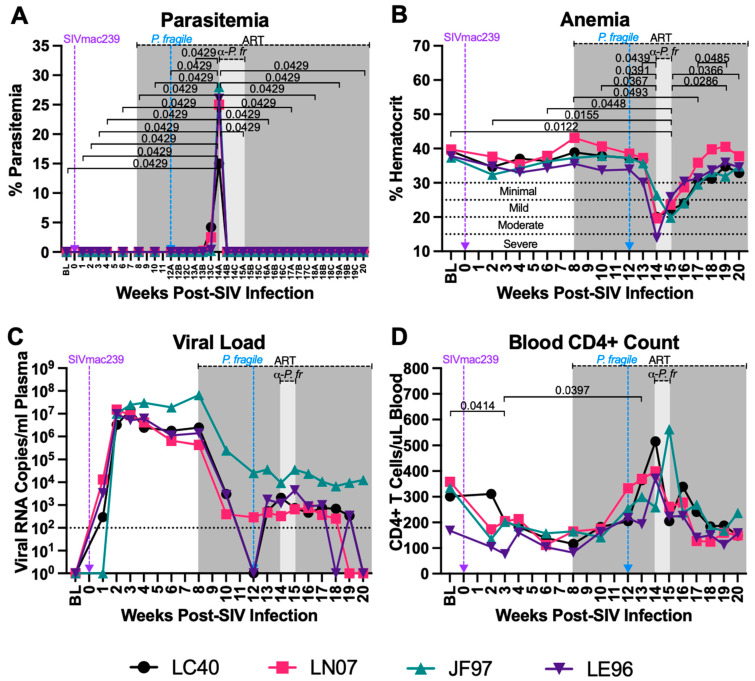
*P. fragile* co-infection results in clinical signs of SIV infection despite persistent, daily ART. Peripheral *P. fragile* parasitemia, anemia, SIVmac239 peripheral-blood viral loads (VLs), and blood CD4+ T-cell counts were assessed in adult, male rhesus macaques (RMs) (n = 4). (**A**) Following *P. fragile* inoculation at week 12 post-SIV infection (p.i.), parasitemia was assessed tri-weekly, indicated as weeks A, B, and C p.i. % Parasitemia was assessed via Giemsa staining of thin blood smears and was defined as the percentage of erythrocytes infected by a parasite among all erythrocytes. (**B**) Anemia was assessed by characterizing % hematocrit, defined as the ratio of red blood cells to total blood. (**C**) Plasma VLs (RNA copies/milliliter plasma) were assessed by qPCR. (**D**) Absolute number of CD4+ T cells per microliter of blood was assessed via flow cytometry. In all panels, each RM is represented by a different symbol and color. Baseline (BL) is the average of data collected at weeks 6, 4, 2, and 0 p.i.. Inoculation with SIVmac239 at week 0 p.i. is indicated by a purple dashed arrow. Inoculation with *P. fragile* at week 12 p.i. is indicated by a blue dashed arrow. Antiretroviral therapy (ART) was initiated at week 8 p.i., indicated by the dark-grey bar. Antimalarial treatment occurred throughout week 14 p.i., indicated by the light-grey bar. Statistical significance between all timepoints was calculated using a mixed-effects analysis with the Geisser–Greenhouse correction and a Tukey’s multiple-comparison test, with individual variances computed for each comparison. Significant multiplicity-adjusted *p* values are shown above horizontal black bars.

Parasitemia and anemia are clinical hallmarks of malaria [75,76]. Following *P. fragile* co-infection, parasitemia was assessed tri-weekly (A, B, C). RMs reached peak parasitemia by week 14A p.i. and had undetectable parasitemia following antimalarial treatment. When comparing all timepoints to each other, we observed that parasitemia was significantly elevated at week 14A p.i. as compared to all other timepoints *(p =* 0.0429; Figure 2A). Similarly, in a follow-up analysis that compared all timepoints to BL only, week 14A p.i. was significantly elevated as compared to BL (*p* = 0.0246; Supplemental Table S3). All RMs experienced mild to severe anemia between weeks 14 and 15 p.i., coinciding with peak parasitemia (Figure 2B). When comparing all timepoints to each other, we observed that percent hematocrit (%HCT) was significantly lower at week 14 p.i. compared to weeks 10, 12, and 13 p.i. (*p* = 0.0367, 0.0391, and 0.049, respectively; Figure 2B). Additionally, %HCT was significantly lower at week 15 p.i. compared to BL and weeks 2, 6, 19, and 20 p.i. (*p* = 0.0122, 0.0155, 0.0448, 0.0286, and 0.0366, respectively; Figure 2B). RMs remained mildly anemic at week 17 p.i., demonstrated by significantly lower %HCTs compared to those at week 8 p.i. (*p* = 0.0493; Figure 2B). By week 18 p.i., all RMs were non-anemic and had significantly greater %HCTs at week 19 p.i. as compared to week 17 p.i. (*p* = 0.0485; Figure 2B). In follow-up analyses, the %HCTs at weeks 4, 14, 15, 16, and 17 p.i. were significant elevated as compared to BL (*p =* 0.0383, 0.0298, 0.0055, 0.027, and 0.037, respectively; Supplemental Table S4). Taken together, *P. fragile* co-infection of ART-treated SIV+ RMs resulted in parasitemia and anemia, which are hallmarks of clinical malaria, indicating that there was no cross-resistance between the two infections, allowing for successful modeling of co-infection in the context of ART.

### 3.3. Clinical Signs of SIV Infection Observed Following P. fragile Co-Infection despite Daily ART

Uncontrolled VLs and decreased CD4+ T-cell counts within a few weeks following infection are hallmarks of pathogenic HIV/SIV [77,78,79,80]. Treatment with ART has been shown to suppress viral replication and restore CD4+ T-cell counts in SIV+ RMs, even at end stage of disease [63,81]. Here, we observed that peak viremia in RMs inoculated with SIVmac239 occurred by week 3 p.i. (median = 1.160 × 10^7^ copies/milliliter; Figure 2C). Lower VLs were observed following ART, with two out of four RMs (LC40 and LE96) exhibiting undetectable VLs by week 12 p.i. (Figure 2C). Following *P. fragile* inoculation, all RMs exhibited VLs above the limit of detection from weeks 13 to 17 p.i. (Figure 2C). A longitudinal assessment by JoinPoint regression demonstrated that following *P. fragile* co-infection, the VLs of all four RMs remained unchanged, indicating viral persistence (Supplemental Figure S3). Between weeks 18 and 20 p.i., one RM (LE96) had transiently detectable VLs, two RMs had undetectable VLs by weeks 19 (LN07) and 20 (LC40) p.i., with the final RM (JF97) remaining incompletely suppressed. When comparing all timepoints to each other, we observed that absolute CD4+ T-cell counts were significantly lower at week 3 p.i. compared to BL (*p* = 0.0414; Figure 2D). CD4+ T-cell counts remained low until ART initiation at week 8 p.i. (Figure 2D). Following *P. fragile* inoculation, CD4+ T-cell counts were significantly increased at week 13 p.i. compared to week 3 p.i. (*p* = 0.0397; Figure 2D). By week 17 p.i. following antimalarial treatment, CD4+ T-cell counts returned to pre-ART levels (Figure 2D). In follow-up analyses comparing all timepoints to BL only, weeks 3 and 8 p.i., timepoints that are representative of early acute and late acute/early chronic SIV infection, were significantly elevated as compared to BL (*p* = 0.019 and 0.0362, respectively; Supplemental Table S4). These data indicate that clinical signs of SIV infection, including VLs above the limit of detection and fluctuations in CD4+ T cell counts, were apparent following *P. fragile* co-infection of ART-treated SIV+ RMs.

### 3.4. Increased Levels of Inflammatory Markers but Unchanged Neutrophil Frequency and Apoptosis Were Observed during ART-Treated SIV/P. fragile Co-Infection

**Figure 3:**
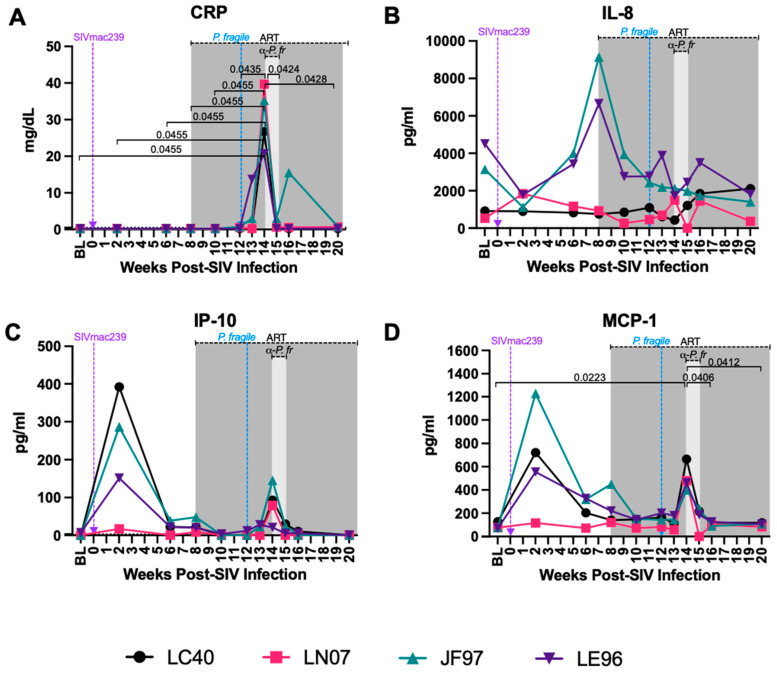
Variable levels of inflammatory markers throughout *P. fragile* co-infection of ART-treated SIVmac239-infected rhesus macaques. CRP, cytokine, and chemokine levels were measured throughout *P. fragile* co-infection of ART-treated SIVmac239-infected rhesus macaques (RMs) (n = 4). (**A**) CRP levels were measured in serum by a Beckman au480. (**B**–**D**) IL-8 (**B**), IP-10 (**C**), and MCP-1 (**D**) levels were measured in plasma by LegendPlex. In all panels, each RM is represented by a different symbol and color. Baseline (BL) is the average of data collected at weeks 6, 2, and 0 post-SIV infection (p.i.). Inoculation with SIVmac239 at week 0 p.i. is indicated by a purple dashed arrow. Inoculation with *P. fragile* at week 12 p.i. is indicated by a blue dashed arrow. Antiretroviral therapy (ART) was initiated at week 8 p.i., indicated by the dark-grey bar. Antimalarial treatment occurred throughout week 14 p.i., indicated by the light-grey bar. Statistical significance between all timepoints was calculated using a mixed-effects analysis with the Geisser–Greenhouse correction and a Tukey’s multiple-comparison test, with individual variances computed for each comparison. Significant multiplicity-adjusted *p* values are shown above horizontal black bars.

CRP is an acute-phase plasma marker of inflammation that has been shown to be elevated in ART-naïve PWH and that is associated with HIV disease progression, even despite ART [82,83,84,85]. Additionally, elevated CRP has been observed in individuals with both complicated and uncomplicated malaria and can be used as a biomarker for monitoring of the malaria severity [86,87]. Here, we observed that serum CRP was significantly increased at week 14 p.i. as compared to BL and weeks 2, 6, 8, and 10 p.i. (*p* = 0.0455 for all), as well as compared to weeks 12, 15, and 20 p.i. (*p* = 0.0435, 0.0424, and 0.0428, respectively; Figure 3A). Similarly, when all timepoints were compared to BL only, CRP was significantly increased at week 14 p.i. as compared to BL (*p =* 0.023; Supplemental Table S5). These data suggest that *P. fragile* infection is linked with elevated CRP expression that coincides with peak parasitemia.

Both HIV and *Plasmodium* infection result in elevated levels of pro-inflammatory cytokines and chemokines [23,88]. Here, we observed some longitudinal and inter-animal variation in plasma levels of interleukin (IL)-8 and interferon gamma-induced protein 10 (IP-10), but no statistically significant changes were observed in these analytes throughout *P. fragile* co-infection of ART-treated SIV+ RMs when all timepoints were compared to each other and when compared to BL only (Figure 3B,C; Supplemental Table S5). Plasma monocyte chemoattractant protein-1 (MCP-1) was significantly increased at week 14 p.i. compared to BL and weeks 16 and 20 p.i. (*p* = 0.0223, 0.0406, and 0.0412, respectively; Figure 3D). When all timepoints were compared to BL only, MCP-1 was significantly increased at week 14 p.i. as compared to BL (*p* = 0.0111; Supplemental Table S5). All other inflammatory analytes remained unchanged over time (Supplementary Figure S4). Given that IP-10 and MCP-1 were higher at peak viremia, followed by a reduction during ART and an increase during *P. fragile* co-infection that coincided with peak parasitemia, these data indicate that elevations in inflammatory cytokines and chemokines may be influenced by SIV infection alone as well as by *P. fragile* co-infection of ART-treated SIV+ RMs.

**Figure 4:**
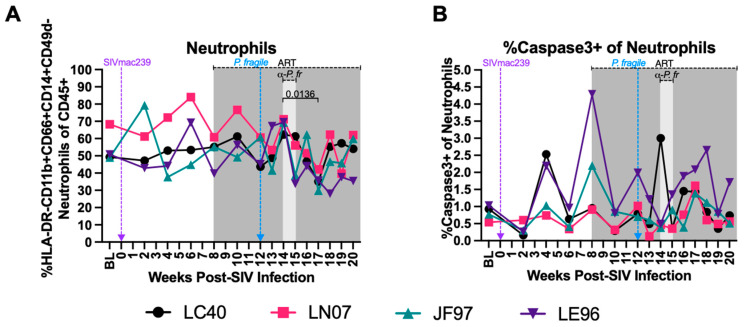
Minimal disruption of peripheral neutrophil frequencies and percentages of neutrophils undergoing apoptosis during *P. fragile* co-infection of ART-treated SIVmac239-infected rhesus macaques. Total neutrophil frequencies and frequencies of neutrophils undergoing apoptosis were assessed in whole blood before and after *P. fragile* co-infection of ART-treated SIVmac239-infected rhesus macaques (RMs) (n = 4) by flow cytometry. (**A**) Neutrophil (HLA-DR-CD11b+CD66abce+CD14+) frequency of live CD45+ cells was assessed throughout co-infection. (**B**) The frequency of neutrophils undergoing apoptosis (caspase3+) was assessed throughout co-infection. In both panels, each RM is represented by a different symbol and color. Baseline (BL) is the average of data collected at weeks 6, 4, 2, and 0 post-SIV infection (p.i.). Inoculation with SIVmac239 at week 0 p.i. is indicated by a purple dashed arrow. Inoculation with *P. fragile* at week 12 p.i. is indicated by a blue dashed arrow. Antiretroviral therapy (ART) was initiated at week 8 p.i., indicated by the dark-grey bar. Antimalarial treatment occurred throughout week 14 p.i., indicated by the light-grey bar. Statistical significance between all timepoints was calculated using a mixed-effects analysis with the Geisser–Greenhouse correction and a Tukey’s multiple-comparison test, with individual variances computed for each comparison. Significant multiplicity-adjusted *p* values are shown above horizontal black bars.

Neutrophil-associated inflammation has been shown to contribute to increased pathogenesis in separate HIV and Plasmodium infection [50,89,90,91,92]. Therefore, we next characterized peripheral neutrophil dynamics and apoptosis via flow cytometry. Neutrophils were identified as viable, CD45+ HLA-DR- CD11b+ CD66abce+ CD14+ CD49d- cells, as previously described (Supplemental Figure S1) [92,93,94,95,96,97]. As previously demonstrated [98], total peripheral neutrophil frequencies were consistent throughout acute SIV infection and ART treatment (Figure 4A). Neutrophil frequencies were significantly decreased following antimalarial treatment as compared to the start of antimalarial treatment (week 17 vs. 14 p.i., *p* = 0.0136), followed by stable neutrophil frequencies until study endpoint in week 20 p.i. (Figure 4A). When all timepoints were compared to BL only, there was a significant difference in the neutrophil frequencies at week 17 p.i. as compared to BL (*p =* 0.0295; Supplemental Table S6). The percentages of apoptotic peripheral neutrophils (caspase3+) fluctuated across all the timepoints (Figure 4B). These data indicate that neutrophil frequencies decrease following clearance of *P. fragile* in ART-treated SIV+ RMs, while neutrophil apoptosis was unchanged throughout ART-treated SIV only infection and *P. fragile* co-infection.

### 3.5. Minimal Disruption in Neutrophil Phagocytosis during ART-Treated SIV/P. fragile Co-Infection

**Figure 5:**
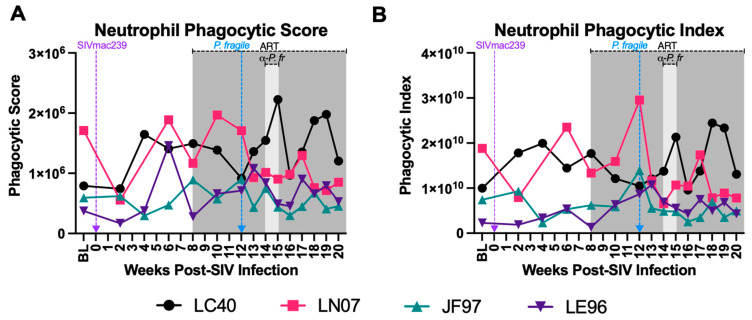
Nominal alterations in neutrophil phagocytosis during *P. fragile* co-infection of ART-treated SIVmac239-infected rhesus macaques. The frequency of phagocytic neutrophils and neutrophil phagocytic capacity were assessed in whole blood throughout *P. fragile* co-infection of ART-treated SIVmac239-infected rhesus macaques (RMs) (n = 4) by flow cytometry. Phagocytosis was determined by assessing the uptake of pHrodo Red *E. coli* bioparticles. (**A**) Neutrophil phagocytic score was calculated by multiplying the absolute number of neutrophils/microliter of whole blood by the percentage of neutrophils positive for the uptake of pHrodo bioparticles. (**B**) Phagocytic index was calculated by multiplying the phagocytic score in (**A**) by the Mean Fluorescence Intensity (MFI) of pHrodo-positive neutrophils. In both panels, each RM is represented by a different symbol and color. Baseline (BL) is the average of data collected at weeks 6, 4, 2, and 0 post-SIV infection (p.i.). Inoculation with SIVmac239 at week 0 p.i. is indicated by a purple dashed arrow. Inoculation with *P. fragile* at week 12 p.i. is indicated by a blue dashed arrow. Antiretroviral therapy (ART) was initiated at week 8 p.i., indicated by the dark-grey bar. Antimalarial treatment occurred throughout week 14 p.i., indicated by the light-grey bar. Statistical significance between all timepoints was calculated using a mixed-effects analysis with the Geisser–Greenhouse correction and a Tukey’s multiple-comparison test, with individual variances computed for each comparison.

Impaired phagocytosis has been observed in separate HIV and *Plasmodium* infection [37,38,45,46,47]. Here, we calculated peripheral blood neutrophil phagocytic score (capability) and index (proficiency) throughout ART-treated SIV/*P. fragile* co-infection. Although inter-animal variations in neutrophil phagocytic score (Figure 5A) and index (Figure 5B) were observed, no statistically significant changes in either phagocytosis parameter were detected over time when comparing all timepoints to each other and when comparing all post-infection timepoints to BL only (Supplemental Table S7).

### 3.6. Decreased Plasma Levels of Neutrophil Granule Components during ART-Treated SIV/P. fragile Co-Infection

**Figure 6:**
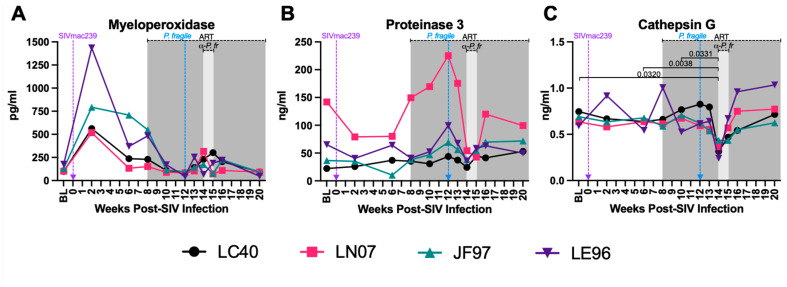
Decreased plasma levels of neutrophil degranulation markers during *P. fragile* co-infection of ART-treated SIVmac239-infected rhesus macaques. Products of neutrophil degranulation were measured throughout *P. fragile* co-infection of ART-treated SIVmac239-infected rhesus macaques (RMs) (n = 4) via ELISA. Myeloperoxidase (**A**), Proteinase 3 (**B**), and Cathepsin G (**C**) levels were measured in plasma by ELISA. In all panels, each RM is represented by a different symbol and color. Baseline (BL) is the average of data collected at weeks 6, 2, and 0 post-SIV infection (p.i.). Inoculation with SIVmac239 at week 0 p.i. is indicated by a purple dashed arrow. Inoculation with *P. fragile* at week 12 p.i. is indicated by a blue dashed arrow. Antiretroviral therapy (ART) was initiated at week 8 p.i., indicated by the dark-grey bar. Antimalarial treatment occurred throughout week 14 p.i., indicated by the light-grey bar. Statistical significance between all timepoints was calculated using a mixed-effects analysis with the Geisser–Greenhouse correction and a Tukey’s multiple-comparison test, with individual variances computed for each comparison. Significant multiplicity-adjusted *p* values are shown above horizontal black bars.

Plasma levels of MPO, PR3, and CATG, three extracellular neutrophil degranulation secreted components [99,100], were quantified throughout ART-treated SIV/*P. fragile* co-infection. Consistent with prior work [101], plasma MPO levels were elevated in all four RMs at week 2 p.i., but no statistically significant differences were observed throughout SIV infection, ART treatment, or *P. fragile* co-infection (Figure 6A). Likewise, plasma levels of PR3 were stable throughout acute SIV infection, ART treatment, and *P. fragile* co-infection (Figure 6B). Finally, a statistically significant decrease in CATG was observed at week 14 p.i., compared to BL and weeks 6 and 10 p.i. (*p* = 0.032, 0.0038, and 0.0331, respectively), followed by a return to BL levels following antimalarial treatment (Figure 6C). When all timepoints were compared to BL alone, plasma levels of CATG were significantly decreased at week 14 p.i. as compared to BL (*p* = 0.0161, Supplemental Table S8). These data indicate that *P. fragile* co-infection of ART-treated SIV+ RMs resulted in lowered plasma levels of CATG.

### 3.7. Increased Plasma Biomarkers of NET Formation during ART-Treated SIV/P. fragile Co-Infection

**Figure 7:**
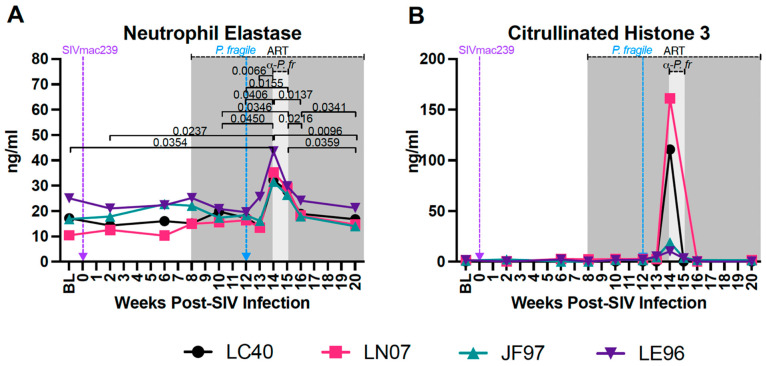
Increased plasma levels of neutrophil extracellular trap markers during *P. fragile* co-infection of ART-treated SIVmac239-infected rhesus macaques. Markers of neutrophil extracellular trap formation were measured throughout *P. fragile* co-infection of ART-treated SIVmac239-infected rhesus macaques (RMs) (n = 4) via ELISA. (**A**) Neutrophil elastase and (**B**) Citrullinated histone 3 levels were measured in plasma by ELISA. In both panels, each RM is represented by a different symbol and color. Baseline (BL) is the average of data collected at weeks 6, 2, and 0 post-SIV infection (p.i.). Inoculation with SIVmac239 at week 0 p.i. is indicated by a purple dashed arrow. Inoculation with *P. fragile* at week 12 p.i. is indicated by a blue dashed arrow. Antiretroviral therapy (ART) was initiated at week 8 p.i., indicated by the dark-grey bar. Antimalarial treatment occurred throughout week 14 p.i., indicated by the light-grey bar. Statistical significance between all timepoints was calculated using a mixed-effects analysis with the Geisser–Greenhouse correction and a Tukey’s multiple-comparison test, with individual variances computed for each comparison. Significant multiplicity-adjusted *p* values are shown above horizontal black bars.

Excessive NET formation contributes to inflammation in separate HIV and *Plasmodium* infection [33,48,52,53]. We assessed plasma levels of NE and CitH3, biomarkers of NET formation [102], throughout ART-treated SIV/*P. fragile* co-infection. Plasma levels of NE were significantly increased at week 14 p.i. compared to BL and weeks 2, 10, 12, and 13 p.i. (*p* = 0.0354, 0.0237, 0.0450, 0.0406, and 0.0066, respectively; Figure 7A) but were significantly reduced at weeks 16 and 20 p.i. as compared to week 14 p.i. (*p* = 0.0137 and 0.0096, respectively) and week 15 p.i. (*p* = 0.0359 and 0.0216, respectively; Figure 7A). Plasma NE was significantly lower at week 20 p.i. compared to week 16 p.i. (*p* = 0.0341; Figure 7A). In follow-up analyses comparing all timepoints to BL only, plasma NE levels were significantly elevated at week 14 p.i. as compared to BL (*p* = 0.0179; Supplemental Table S9), highlighting the association between co-infection and elevated NE expression. Plasma levels of CitH3 were elevated in all four RMs at week 14 p.i., but no statistically significant differences were observed throughout SIV infection, ART treatment, or *P. fragile* infection (Figure 7B). In sum, these findings suggest that *P. fragile* infection is specifically linked with increased levels of NE, a marker of NET formation.

### 3.8. Increased Plasma Markers of Gut Permeability and Microbial Translocation during ART-Treated SIV/P. fragile Co-Infection

**Figure 8:**
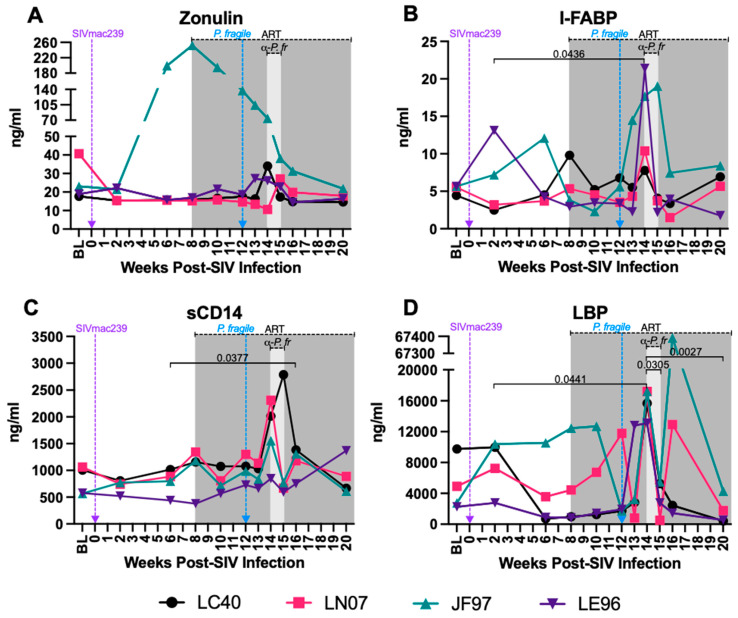
Increased levels of microbial translocation and gastrointestinal (GI) barrier permeability markers during *P. fragile* co-infection of ART-treated SIVmac239-infected rhesus macaques. Markers of microbial translocation and GI barrier permeability were measured throughout *P. fragile* co-infection of ART-treated SIVmac239-infected rhesus macaques (RMs) (n = 4) via ELISA. Zonulin (**A**), intestinal fatty acid-binding protein (I-FABP; **B**), Soluble CD14 (sCD14; **C**), and LPS-binding protein (LBP; **D**) levels were measured in plasma by ELISA. In all panels, each RM is represented by a different symbol and color. Baseline (BL) is the average of data collected at weeks 6, 2, and 0 post-SIV infection (p.i.). Inoculation with SIVmac239 at week 0 p.i. is indicated by a purple dashed arrow. Inoculation with *P. fragile* at week 12 p.i. is indicated by a blue dashed arrow. Antiretroviral therapy (ART) was initiated at week 8 p.i., indicated by the dark-grey bar. Antimalarial treatment occurred throughout week 14 p.i., indicated by the light-grey bar. Statistical significance between all timepoints was calculated using a mixed-effects analysis with the Geisser–Greenhouse correction and a Tukey’s multiple-comparison test, with individual variances computed for each comparison. Significant multiplicity-adjusted *p* values are shown above horizontal black bars.

The loss of the GI epithelial barrier integrity leads to microbial translocation in both HIV and *Plasmodium* infection [39,43,44,103]. Here, we examined plasma levels of zonulin, a protein that modulates tight junctions [104]; I-FABP, a circulating biomarker of intestinal injury [105,106]; sCD14, which is released from monocytes upon lipopolysaccharide (LPS) stimulation; and LBP, which assists in LPS recognition by interacting with LPS receptors [107]. Inter-animal variation in plasma zonulin levels was observed throughout the study (Figure 8A). Plasma I-FABP was significantly increased at week 14 p.i. compared to week 2 p.i. (*p* = 0.0436; Figure 8B), sCD14 was significantly greater at week 16 p.i. compared to week 6 p.i. (*p* = 0.0377; Figure 8C), and LBP was significantly increased at week 14 p.i. compared to weeks 2, 15 and 20 p.i. (*p* = 0.0441, 0.0305, and 0.0027, respectively; Figure 8D). In follow-up analyses comparing all timepoints to BL only, plasma LBP at week 14 p.i. was significantly increased as compared to BL (*p =* 0.0367; Supplemental Table S10). These findings indicate that *P. fragile* co-infection may exacerbate GI epithelial barrier disruption, resulting in microbial translocation, in ART-treated SIV+ RMs.

### 3.9. Markers of SIV and P. fragile Infection, Neutrophil Frequency and Function, Inflammation, GI Permeability, and Microbial Translocation Are Correlated in P. fragile Co-Infected ART-Treated SIV+ RMs

**Figure 9:**
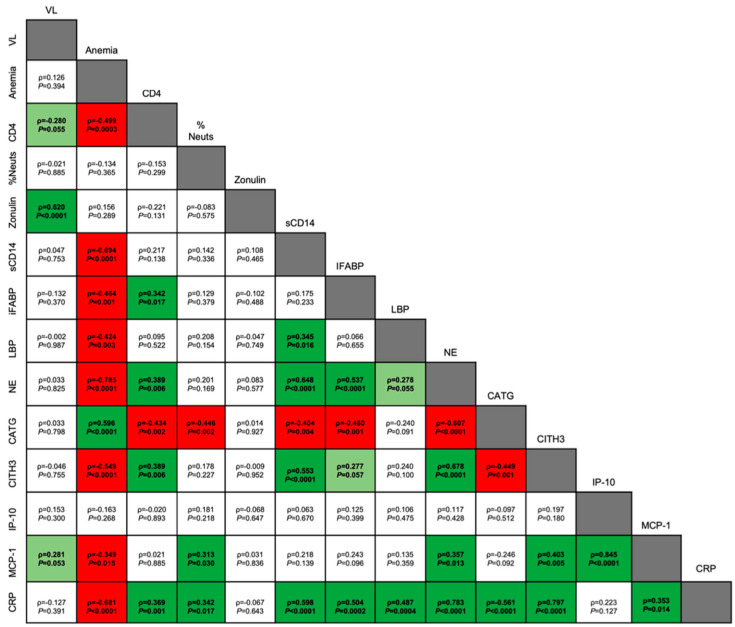
Multivariate ANOVA (MANOVA) reveals significant correlations between clinical markers of SIV and malaria infection, as well as neutrophil frequency and function and peripheral markers of GI dysfunction. Pearson’s partial correlation coefficients were generated using a MANOVA for 14 different parameters (VL; anemia; absolute CD4+ count; peripheral neutrophils; plasma zonulin; sCD4; I-FABP; LBP; NE; cathepsin G; CitH3; IP-10; MCP-1; and CRP). The correlation coefficients were adjusted against animal number. Boxes highlighted in light green represent positive correlations trending towards significance (0.05 < *p* < 0.07), and boxes highlighted in dark green or red represent statistically significant positive and negative correlations, respectively (*p* < 0.05).

To identify links between markers of SIV and *P. fragile* infection, neutrophil frequency and function, inflammation, GI barrier permeability, and microbial translocation, we conducted a MANOVA, controlling for time (Figure 9). SIV VLs were positively associated with plasma zonulin, a marker of gut dysfunction ([ρ *=* 0.620, *p* < 0.0001]; Figure 9). Absolute CD4+ T-cell counts were positively associated with markers of NET formation (NE and CitH3, both ρ = 0.389, *p* = 0.006), gut dysfunction (I-FABP [ρ *=* 0.342, *p* = 0.017]), and systemic inflammation (CRP [ρ = 0.369, *p* = 0.001]) but were negatively associated with degranulation (CATG [ρ *=* −0.434, *p* = 0.002]; Figure 9). % HCT was positively associated with markers of degranulation (CATG [ρ = −0.576, *p* < 0.0001]) but was negatively associated with markers of NET formation (NE [ρ *=* −0.785, *p* < 0.0001] and CitH3 [ρ = −0.549, *p* < 0.0001]), chemokine production (MCP-1 [ρ = −0.349, *p* = 0.015]), GI dysfunction (sCD14 [ρ *=* −0.694, *p* < 0.0001], I-FABP [ρ *=* −0.464, *p* = 0.001], LBP [ρ = −0.424, *p* = 0.003]), and systemic inflammation (CRP [ρ = −0.681, *p* < 0.0001]). Neutrophil frequency was positively associated with chemokine production (MCP-1, [ρ = 0.313, *p* = 0.030]) and systemic inflammation (CRP [ρ = 0.342, *p* = 0.017]) but was negatively associated with markers of degranulation (CATG [ρ = −0.514, *p* < 0.0002]). Chemokine production (MCP-1) was positively associated with markers of NET formation (NE [ρ = 0.357, *p* = 0.013], CITH3 [ρ = 0.403, *p* = 0.005]), and other markers of inflammation (IP-10 [ρ = 0.845, *p* < 0.0001] and CRP [ρ = 0.353, *p* = 0.014]; Figure 9). Markers of GI dysfunction (sCD14 and IFABP) were positively correlated with markers of NET formation (NE [ρ = 0.648, *p* < 0.0001] and ρ = 0.537, *p* < 0.0001]), respectively, and CITH3 ([ρ = 0.553, *p* < 0.0001] and [ρ = −0.277, *p* = 0.057], respectively) but were negatively correlated with markers of degranulation (CATG [ρ = −0.404, *p* = 0.004] and [ρ = −0.460, *p* = 0.001], respectively; Figure 9). Markers of GI dysfunction (sCD14, IFABP, and LBP) were also positively associated with a marker of systemic inflammation (CRP [ρ = 0.598, *p* < 0.0001], [ρ = 0.504, *p* = 0.0002], and [ρ = 0.487, *p* = 0.0004], respectively; Figure 9). Finally, a marker of systemic inflammation (CRP) was positively associated with markers of NET formation (NE [ρ = 0.797, *p* < 0.0001] and CitH3 [ρ = 0.783, *p* < 0.0001], respectively) but was negatively correlated with markers of degranulation (CATG [ρ = −0.561, *p* < 0.0001]; Figure 9). These data indicate that *P. fragile* co-infection of ART-treated SIV+ RMs was associated with markers of NET formation, CD4+ T-cell proliferation, inflammation, and chemokine production, which may have allowed for viral persistence and exacerbation of GI barrier disruption and microbial translocation.

## 4. Discussion

In this pilot study, we characterized the impact of *P. fragile* co-infection on ART-treated, SIV+ RMs. Pathogenic SIV infection in RMs has been well-characterized, with RMs exhibiting uncontrolled VLs and decreased CD4+ T-cell counts within two weeks following SIV infection, similar to pathogenic HIV infection in humans [78,79,80]. Additionally, previous work has shown that ART initiation during SIV infection results in decreased VLs within two weeks of starting treatment [63,108], and that ART treatment rapidly restores CD4+ T-cells and T-cell functionality, even at end stage disease [81]. Consistent with these prior findings, all four RMs in our study exhibited elevated VLs and depleted CD4+ T-cell counts during acute SIV infection, followed by decreased VLs and elevated CD4+ T-cell counts post-ART initiation, indicating that all RMs followed the expected progression of acute SIV infection and response to suppressive therapy.

Co-infection of SIV+ RMs with *P. fragile* has been conducted previously [56,57]. However, our pilot study represents the first assessment of *P. fragile* co-infection in the context of ART-treated SIV infection. Here, we observed that ART-treated SIV+ RMs exhibited peripheral parasitemia within two weeks of *P. fragile* co-infection, which coincided with mild–severe anemia. These findings are in line with previous work that demonstrated that RMs inoculated with *P. fragile* via a similar route, but with differences in the dose and treatment regimen used, also exhibited peripheral parasitemia and anemia within two weeks of inoculation [56]. Notably, ART-treated SIV/*P. fragile*-co-infected RMs in our pilot study experienced parasitemia and anemia greater than that observed during *P. fragile* infection only RMs in the previously published study [56], possibly suggesting that *P. fragile* infection in ART-treated SIV+ RMs results in the exacerbation of clinical hallmarks of malaria infection, as compared to *P. fragile* infected only RMs. However, given the differences in the inoculation doses and treatment regimens used in our pilot study and in this previously published work, we are unable to definitively make this conclusion with our current data.

Following *P. fragile* co-infection, SIV+ RMs maintained detectable VLs and decreased CD4+ T-cell counts for several weeks. This observation is in agreement with prior reports noting detectable VLs during co-infection of ART-naïve RMs [56]. Additionally, *Plasmodium* co-infection resulted in increased HIV replication sans ART in vitro and in vivo in humans [9,10,11,109,110,111]. Of note, in this pilot study, one RM (JF97) had persistently high VLs despite ART, as well as higher levels of inflammatory cytokines and chemokines. Previous work has shown associations between host genetics, including the expressions of particular MHC alleles, and high VLs and rapid disease progression in both humans [112] and macaques [113,114]. Recent work suggests an association between Mamu-B*012 and high VLs in RMs, but only with specific KIR alleles [113]. Here, one animal (LE96) did express Mamu-B*012, but KIR genotyping was not performed, and this animal did not exhibit exceptionally high VLs. In addition, there were no marked differences in the clinical history, such as in values reported in the weekly CBCs, blood chemistries, or physical exams between JF97 and the other three RMs assessed here. Additional work is needed to understand JF97′s inability to virally control despite ART. Importantly, there is no expected interaction between the ART regimen (TDF/FTC/DTG) and antimalarial drugs (chloroquine and quinine sulfate) used, indicating that drug–drug interactions are unlikely to be the cause of persistent SIV VLs despite ART treatment [115].

Systemic inflammation is a hallmark of HIV infection, even with consistent ART [24]. Indeed, CRP, an acute-phase marker of inflammation, has been shown to be elevated in ART-naïve and ART-treated PWH [82,83,84,85]. Additionally, increased CRP has been used as a biomarker of malaria severity [86,87]. In our pilot study, we observed increased levels of serum CRP that coincided with peak parasitemia, indicating that *P. fragile* exposure is linked with increased systemic inflammation. We also observed that serum CRP was significantly positively correlated with not just neutrophil frequency but also with markers of NE and CitH3, biomarkers of NET formation [102]. Notably, neutrophil-associated inflammation contributes to pathogenesis during separate HIV and *Plasmodium* infection [50,89,90,91,92]. Prior work has also identified links between residual viral replication during ART and uncontrolled inflammation [116,117]. Thus, neutrophil-associated systemic inflammation could constitute a mechanism underlying continued SIV replication during *P. fragile* co-infection despite ART.

In this pilot study, ART-treated SIV/*P. fragile* co-infection resulted in increased MCP-1, a potent monocyte chemoattractant produced by many cells, including neutrophils [118]. Additionally, we observed significant correlations between plasma MCP-1 with NE and CitH3. Previously, increased MCP-1 was associated with NET release in individuals with myocardial infarction, which, in turn, stimulates further MCP-1 production [119]. Taken together, increased MCP-1 production during ART-treated SIV/*P. fragile* co-infection could result from increased NET formation [120], and these processes may cooperatively contribute to heightened inflammation, allowing for persistent viral replication. Supporting this, plasma MCP-1 was correlated with SIV VL, indicating a potential association between neutrophil-mediated inflammation and SIV reactivation during *P. fragile* co-infection.

Notably, although neutrophil frequency was minimally altered throughout our ART-treated SIV/*P. fragile* co-infection pilot study, a significant shift in NE, a peripheral marker of neutrophil function, was detected. We identified that the increase in this marker of NET formation was significantly inversely correlated with anemia, a clinical marker of *Plasmodium* infection. Conversely, we noted decreased expression of the neutrophil degranulation marker CATG and unchanged neutrophil phagocytosis. Neutrophil selection between defense mechanisms appears to be size-dependent: the phagocytosis of smaller microbes inhibits NET release, but inhibition of phagocytosis due to microbe size prompts NETosis [121]. Notably, previous studies have shown that both opsonized and non-opsonized monocyte/macrophage phagocytoses of *P. falciparum*-infected erythrocytes are impaired in PWH in vitro and in vivo [121,122,123], although non-opsonized parasite phagocytosis was restored after 6 months of ART [123]. Our pilot data could therefore indicate that an insufficient phagocytic response during SIV/*P. fragile* co-infection skews neutrophils towards NET formation, providing a potential mechanism by which they contribute to systemic inflammation during ART-treated SIV/*P. fragile* co-infection.

GI dysfunction is a major pathogenic process in separate HIV and malaria infection [42,43,44,124]. *Plasmodium* parasite sequestration in the GI tract causes barrier permeability [103,125], while HIV-associated GI mucosal dysfunction is linked with the loss of barrier integrity and elevated microbial translocation [43,107]. Our data indicating that SIV VL is positively correlated with plasma zonulin levels are in agreement with this. Importantly, GI neutrophil infiltration and survival have previously been correlated with HIV-associated GI mucosal dysfunction [39,40,41]; thus, GI neutrophil activity in response to parasite sequestration could further exacerbate SIV-associated GI dysfunction. Our data indicating that plasma markers of GI barrier permeability (plasma sCD14, iFABP, LBP) were associated peripheral markers of NET formation (plasma NE and CitH3) in ART-treated SIV/*P. fragile*-co-infected RMs support this. Moreover, our observation of an inverse correlation between sCD14, LBP, and anemia, a hallmark of clinical malaria infection, further supports a potential relationship between malaria-induced GI dysfunction, possibly mediated by GI neutrophil infiltration and inflammation, during ART-treated SIV co-infection. A caveat of these data is that they are currently limited to plasma markers of neutrophil function, GI barrier integrity, and microbial translocation. Future studies will focus on assessing the relationship between these factors in mucosal tissues to fully define the mechanistic relationships between neutrophil-associated GI dysfunction and SIV/malaria co-infection pathogenesis.

A major strength of our work is the utilization of an NHP model that mimics HIV and *P. falciparum* infection [54,55,69,70]. Additionally, our longitudinal assessments provided an opportunity to identify how *Plasmodium* co-infection could influence SIV pathology in the setting of viral suppression. Caveats of our study are the short duration of ART and the lack of complete viral suppression for an extended period prior to *P. fragile* co-infection. Our rationale for initiating the ART at week 8 p.i. was to allow RMs to establish an early viremic setpoint, representative of late acute/early chronic infection, which has previously been reported to occur as early as 42 days post-infection for SIVmac239 [64,65,66]. While previous work has shown that ART initiation in SIV+ RMs results in decreased viremia within two weeks of initiation, in some cases it may take longer to achieve consistently undetectable VLs [63,108]. In addition, while current guidelines recommend that individuals with recent HIV infection, defined as the ≤ 6-month period after infection when anti-HIV antibodies are detectable, begin ART as soon as possible, there are often delays in ART initiation post-diagnosis [67,68,126]. This means that even with access to routine, point-of-care HIV testing and same-day ART initiation, it is likely that most individuals will have had time for HIV pathogenesis to be established prior to ART initiation. Therefore, considering these data in both humans and NHPs, in our pilot study, we elected to initiate ART at 8 weeks p.i. in order to balance the goals of establishing pathogenic SIV infection prior to ART initiation and modeling a scenario of early diagnosis and ART treatment. These choices, along with the pilot nature of our study, mean that we are limited in the scope of the conclusions that can be made in regard to the impact of *P. fragile* co-infection during fully ART-suppressed SIV infection. Future studies to address this limitation by extending the amount of time that RMs are on ART to allow for full viral suppression prior to co-infection are warranted. Additionally, it may be possible to use this model to iteratively test the impact of co-infection before and after full viral suppression, with the goal of elucidating the impact of *Plasmodium* infection at different stages following ART initiation on pathology, disease outcome, and ART efficacy.

It is important to note that in malaria-endemic areas, there is the potential for recurrent *Plasmodium* spp. infections. Recurrent infections are defined as newly detectable blood-stage parasitemia after the clearance of a previous infection [127]. In the context of *P. falciparum*, recurrent infections can occur either due to re-infection via a new mosquito bite or recrudescence associated with sub-patent parasitemia that was previously undetectable [128]. Given the pilot nature of our study, here, we elected to examine the effects of a single *P. fragile* exposure in ART-treated SIV+ RMs. However, by establishing the utility of this NHP model of *P. fragile* co-infection in the context of ART-treated SIV infection, future work exploring recurrent *P. fragile* infection in SIV+ RMs will be possible and important to gain a full understanding of the increased disease pathogenesis that occurs during co-infection.

Another caveat to this pilot study is the usage of chloroquine and quinine sulfate as antimalarial treatments. Both quinine sulfate and chloroquine have been shown to influence various immune parameters, including NET formation and phagocytosis [129,130,131,132,133]. Given that our data indicate that neutrophil phagocytosis was unchanged throughout antimalarial drug treatment, and because the significant increase we observed in NE occurred prior to quinine sulfate and chloroquine administration, the use of these drugs in our study likely did not impact our observations on the effects of *P. fragile* co-infection on neutrophil responses in ART-treated SIV+ RMs. Nonetheless, we cannot completely discount the possibility that quinine sulfate and/or chloroquine administration may have affected these parameters, and future studies should include antimalarial-drug-treatment-only groups in not just healthy RMs but also in SIV+ and ART-treated SIV+ RMs to control for this possibility.

The work presented here is from a pilot study; thus, an inherent limitation is the small number of RMs used, particularly since inter-animal variation was observed in some parameters, which may have contributed to the lack of statistical significance observed at some timepoints. Additional work with more animals, with the addition of matched, contemporary ART-treated SIV-only, *P. fragile*-only, and ART and antimalarial treatment only control groups, will be necessary to fully characterize the kinetics and impact of co-infection.

Here, we primarily focused on the peripheral innate immune response and neutrophils in particular. Future work on mucosal tissues, including immune cell enumeration and immunophenotyping in the context of ART-treated SIV/*P. fragile* co-infection, is needed. Moreover, work to determine the role of additional innate immune cells, such as monocytes and macrophages, and their interactions with adaptive immune cells, such as CD4 T-cells and their subsets, is warranted. For example, Th17 CD4 T-cells have been shown to play a key role in maintaining GI homeostasis and have been shown to recruit neutrophils to sites of inflammation and mucosal injury [134,135,136]. Thus, an important future direction will be to assess neutrophil/Th17 cell dynamics both in the periphery and directly in mucosal tissue to determine how these interactions impact GI impairment and damage in the context of SIV/*P. fragile* co-infection.

Finally, in this pilot study, we elected to infect RMs with *P. fragile* via i.v. inoculation with blood-stage parasites. This means that the *P. fragile* infection in our model bypassed the clinically silent liver stage of *Plasmodium* infection. Previous work has found that although neutrophil frequency and function are unchanged during the pre-erythrocytic stage of *Plasmodium* infection, total leukocytes are significantly increased [137]. Future studies that incorporate *Plasmodium* transmission through infected mosquito bites will be more reflective of vector parasite transmission and allow for the assessment of the pre-erythrocytic stage of *Plasmodium* infection.

In this present study, we focused on establishing the SIV/*P. fragile* co-infection model in the context of ART, in an effort to validate the utility of our model and guide its use in future explorations. The pilot data presented here indicate that ART-treated SIV/*P. fragile*-co-infected RMs displayed clinical signs of SIV and malaria, which were associated with shifts in neutrophil function and increased markers of GI mucosal dysfunction. These observations could have implications for HIV and malaria co-endemic areas. *Plasmodium* co-infection in PWH may lead to viral reactivation, creating a scenario in which the rates of HIV transmission are sustained even despite widespread use and adherence to ART. Indeed, PWH who have viral loads greater than 1000 copies/milliliter, regardless of ART, are at an increased risk of transmitting HIV [138]. It is important to note, however, that little work has been conducted to examine whether a sustained SIV VL correlates with an increased risk of SIV transmission; thus, additional work will be needed to extend our observations using an NHP model to ART-treated PWH with *Plasmodium* co-infection. Moreover, our observations of a link between neutrophil function and clinical signs of SIV/*P. fragile* and GI dysfunction highlight the need for additional research to define the role of this cellular subset during co-infection and supports the rationale for examining the potential of neutrophil-targeted interventions to reduce the burden of HIV and malaria, separately and in the context of co-infection. In summary, the pilot data presented here support the utility of this NHP model of ART-treated SIV/*P. fragile* co-infection to answer pertinent questions about the impact of co-infection on immunity and disease outcomes.

## Data Availability

All data are available within the manuscript or in its Supplemental Materials.

## Supplementary Materials

The following supporting information can be downloaded at: https://www.mdpi.com/article/10.3390/v16071036/s1, Supplemental Figure S1: Representative flow plots demonstrating gating strategy used to identify and phenotype neutrophils; Supplemental Figure S2: Representative flow plots demonstrating gating strategy used to evaluate phagocytosis via uptake of pHrodo particles; Supplemental Figure S3: JoinPoint regression of viral loads generates four distinct slopes; Supplemental Figure S4: Peripheral cytokines and chemokines; Supplemental Table S1: Neutrophil flow cytometry antibodies; Supplemental Table S2: Absolute count flow cytometry antibodies; Supplemental Table S3: Median (95% CI) and *p* values for statistical analysis of differences in parasitemia between baseline and subsequent timepoints; Supplemental Table S4: Median (95% CI) and *p* values for statistical analysis of differences in anemia, viral load, and blood CD4+ T cell count between baseline and subsequent timepoints; Supplemental Table S5: Median (95% CI) and *p* values for statistical analysis of differences in peripheral CRP, IL-8, IP-10, and MCP-1 levels between baseline and subsequent timepoints; Supplemental Table S6: Median (95% CI) and *p* values for statistical analysis of differences in the frequency of total neutrophils and caspase3+ neutrophils between baseline and subsequent timepoints; Supplemental Table S7: Median (95% CI) and *p* values for statistical analysis of differences in the neutrophil phagocytic score and index between baseline and subsequent timepoints; Supplemental Table S8: Median (95% CI) and *p* values for statistical analysis of differences in plasma levels of myeloperoxidase, proteinase 3, and cathepsin G between baseline and subsequent timepoints; Supplemental Table S9: Median (95% CI) and *p* values for statistical analysis of differences in neutrophil elastase and citrullinated histone 3 between baseline and subsequent timepoints; Supplemental Table S10: Median (95% CI) and *p* values for statistical analysis of differences in plasma levels of zonulin, intestinal fatty acid binding protein, soluble CD14, and lipopolysaccharide binding protein between baseline and subsequent timepoints.
