# Supplementary material for: Elevated Inflammation Associated with Markers of Neutrophil Function and Gastrointestinal Disruption in Pilot Study of Plasmodium fragile Co-Infection of ART-Treated SIVmac239+ Rhesus Macaques
Source: Viruses. 2024 Jun 27;16(7):1036. doi: 10.3390/v16071036 (PMC11281461; doi:10.3390/v16071036)
Supplement: Supplementary file 1 [file viruses-16-01036-s001.zip › viruses-3037028-supplementary.pdf]

Supplemental Figure S1

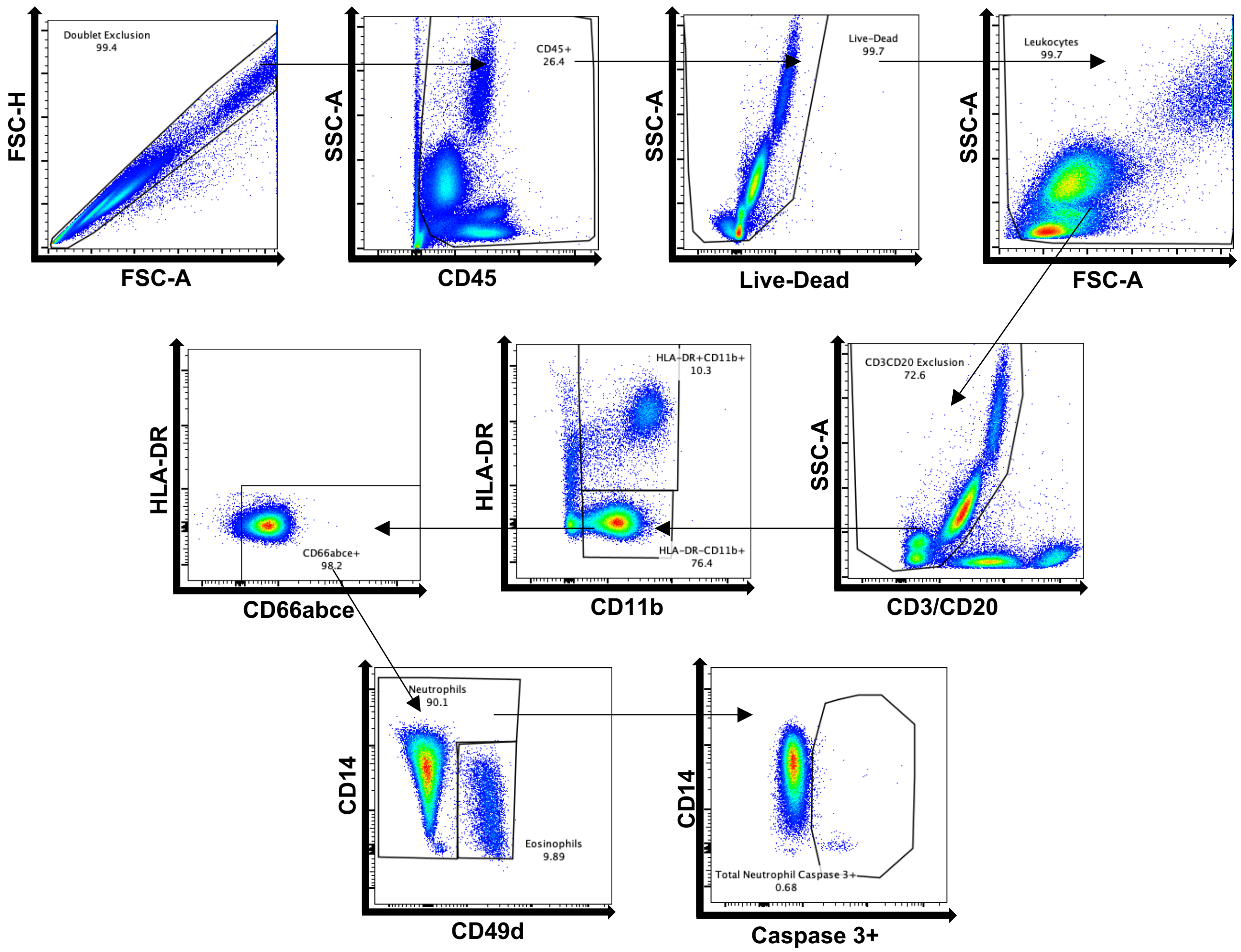

Supplemental Figure S1. Representative flow plots demonstrating gating strategy used to identify and phenotype neutrophils. Multicolor flow cytometry was used to identify neutrophils in whole blood. Depicted here are representative plots from a rhesus macaque (LC40) prior to SIVmac239 inoculation (week -2). Doublets were first excluded using forward scatter (FSC) area and height (FSC-A and FSC-H, respectively) properties. Next, CD45+ cells were identified, dead cells were excluded using an Aqua Live/Dead viability dye, and remaining debris was removed using FSC and side scatter (SSC) properties. T cells and B cells were excluded by gating on CD3- and CD20- cells. Next, granulocytes were identified as HLA-DR- CD11b+ cells. Among total granulocytes, neutrophils and eosinophils were identified as CD66abce+ cells, and then further classified as neutrophils (CD14+ CD49d-) and eosinophils (CD14dim CD49d+). Finally, caspase3+ neutrophils were identified within total neutrophils.

Supplemental Figure S2

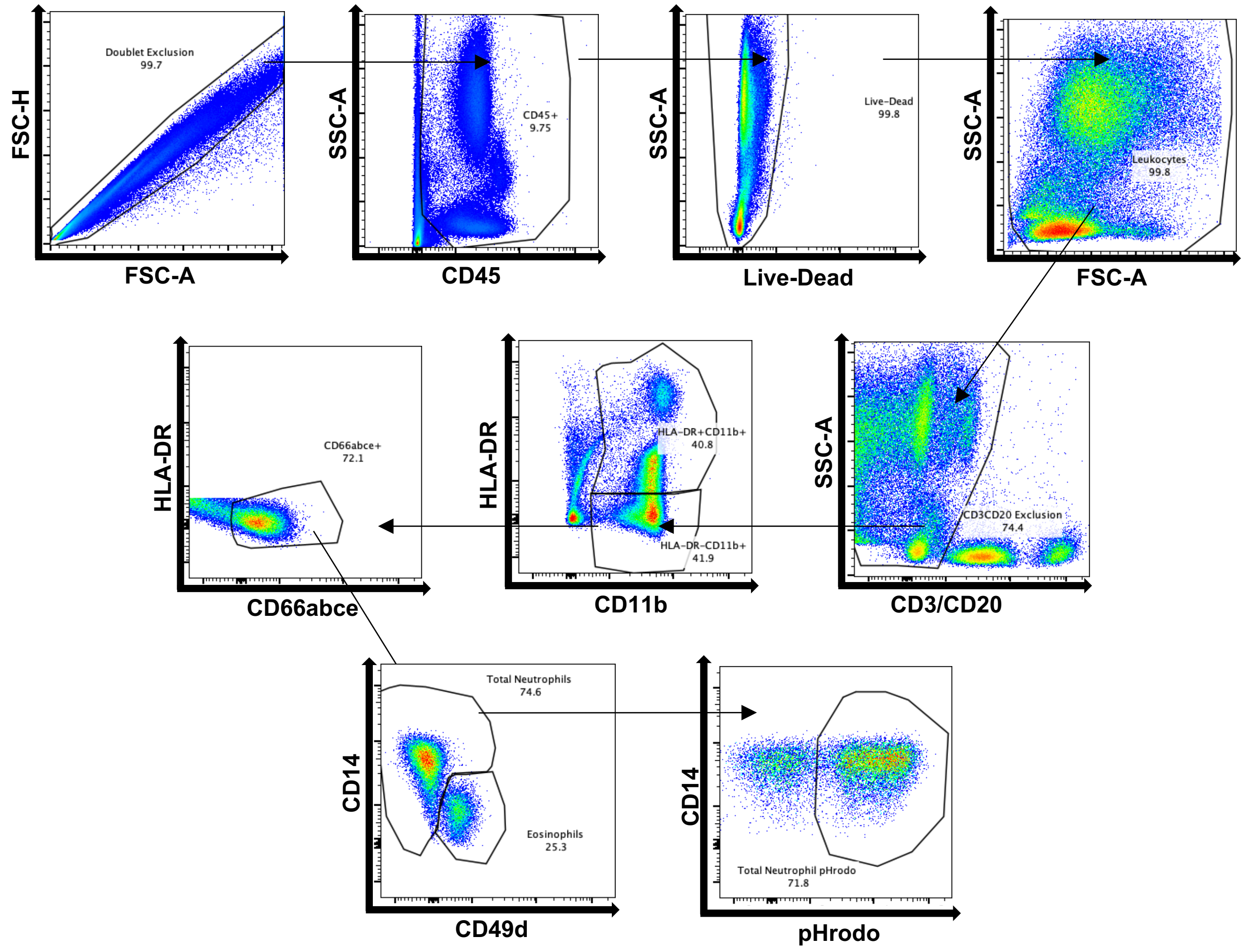

Supplemental Figure S2. Representative flow plots demonstrating gating strategy used to evaluate phagocytosis via uptake of pHrodo particles. Multicolor flow cytometry was used to identify neutrophils that had phagocytosed pHrodo particles. Depicted here are representative plots from a rhesus macaque (LC40) prior to SIVmac239 inoculation (week -2). Doublets were first excluded using forward scatter (FSC) area and height (FSC-A and FSC-H, respectively) properties. Next, CD45+ cells were identified, dead cells were excluded using an Aqua Live/Dead viability dye, and remaining debris was removed using FSC and side scatter (SSC) properties. T cells and B cells were excluded by gating on CD3- and CD20- cells. Next, granulocytes were identified as HLA-DR- CD11b+ cells. Among total granulocytes, neutrophils and eosinophils were identified as CD66abce+ cells, then further classified as neutrophils (CD14+ CD49d-) and eosinophils (CD14dim CD49d+). Phagocytosing neutrophils were defined as pHrodo+ neutrophils among total neutrophils.

Supplemental Figure S3

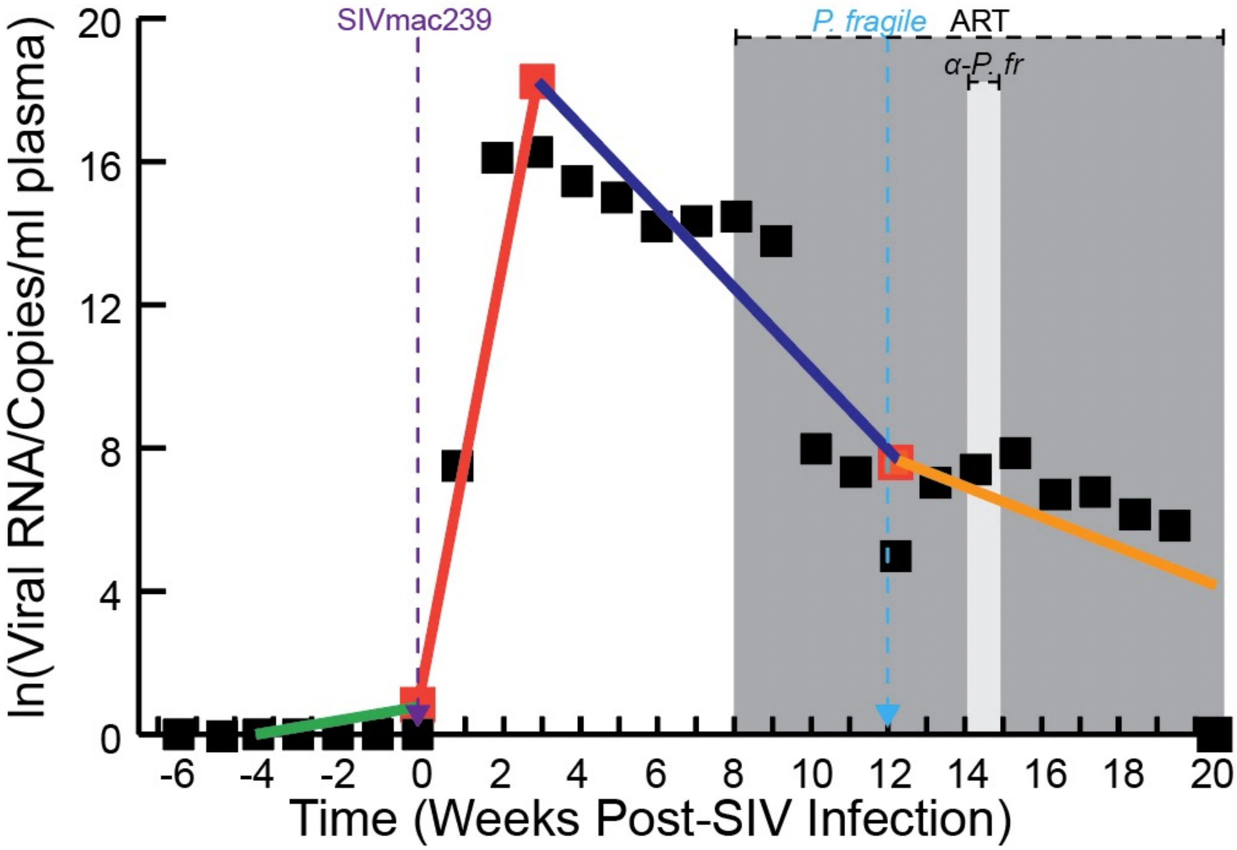

Supplemental Figure S3. JoinPoint regression of viral loads generates four distinct slopes. SIVmac239 peripheral viral loads (VL) were assessed in adult male rhesus macaques (RMs; n=4). When applied to VL data, the JoinPoint Regression Program generated four distinct, statistically significant slopes that corresponded with (1) BL (green), (2) acute SIV infection (red), (3) chronic SIV and ART-treated SIV infection (purple), and (4) *P. fragile*/SIV co-infection (orange). Timepoints in slopes (1) and (4) were statistically insignificant from each other when compared within each slope, indicating persistently unchanged VL values during these phases. Timepoints in slopes (2) and (3) were statistically significantly increased and decreased, respectively, indicating positive and negative changes in VL values (P<0.5 for both).

Supplemental Figure S4

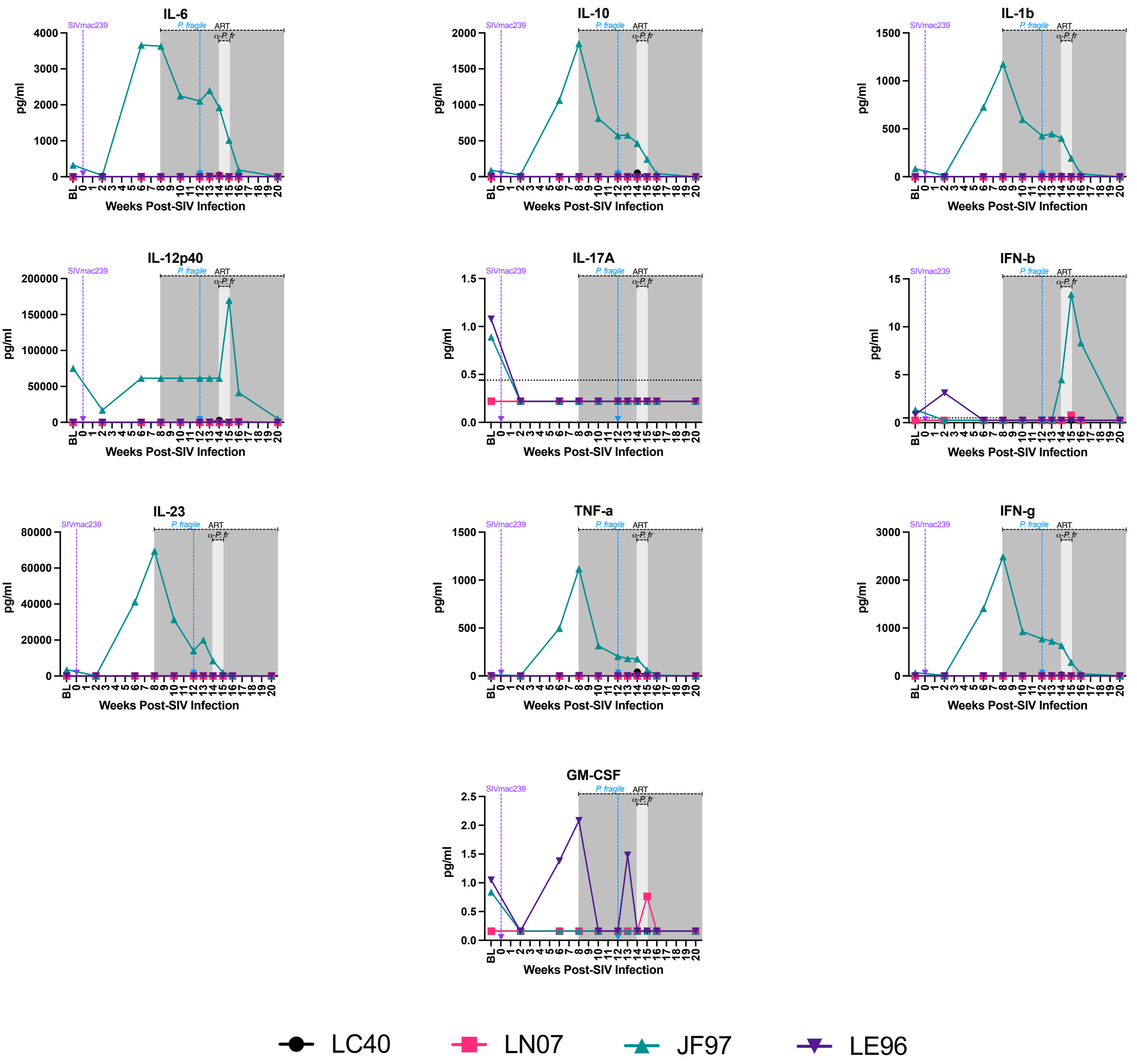

Supplemental Figure S4. Peripheral cytokines and chemokines. Cytokine and chemokine levels were measured throughout *P. fragile* co-infection of ART-treated SIVmac239-infected rhesus macaques (RMs; n=4) via a bead-based multiplex assay (LegendPlex). Analytes measured included IL-6, IL-10, IL-1b, IL-12p40, IL-17A, IFN-b, IL-23, TNF-a, IFN-g, and GM-CSF. In all panels, each RM is represented by a different symbol and color. Baseline (BL) is an average of data collected at weeks -6, -2, and 0 post-SIV infection (p.i.). Inoculation with SIVmac239 at week 0 p.i. is indicated by a purple dashed arrow. Inoculation with *P. fragile* at week 12 p.i. is indicated by a blue dashed arrow. Antiretroviral therapy (ART) was initiated at week 8 p.i., indicated by the dark grey bar. Anti-malarial treatment occurred throughout week 14 p.i., indicated by the light grey bar.

Supplemental Table S1. Neutrophil Flow Cytometry Antibodies

Antibody[1]	Clone	Color	Company	Catalogue Number	Host	Staining Step
CD3	Sp34	FITC	BD Biosciences	556611	Mouse IgG3, λ	Extracellular
CD20	2H7	FITC	BioLegend	302304	Mouse IgG2b, κ	Extracellular
CD66abce	TET2	PerCP-Vio700	Miltenyi	130-119-850	Mouse IgG2b, κ	Extracellular
CD49d	HP2/1	APC	Beckman Coulter	B01682	Mouse IgG1	Extracellular
CD11b	ICRF44	APC-Cy7	BD Biosciences	557754	Mouse IgG1, κ	Extracellular
HLA-DR	G46-6	BV785	BD Biosciences	564041	Mouse IgG2a, κ	Extracellular
CD14	M5E2	PE-Cy5	BioLegend	301864	Mouse IgG2a, κ	Extracellular
CD45	D058-1283	BUV395	BD Biosciences	564099	Mouse IgG1, κ	Extracellular
Caspase3	CD92-605	BV421	BD Biosciences	560627	Rabbit IgG	Intracellular

[1]Antibody name, clone, color, company, catalogue number, and host of extracellular antibodies used in flow cytometric staining to identify neutrophils, neutrophil apoptosis and neutrophil phagocytosis in whole blood.

Supplemental Table S2. Absolute Count Flow Cytometry Antibodies

Antibody[1]	Clone	Color	Company	Catalogue Number	Host	Staining Step
CD3	SP34	FITC	BD Biosciences	556611	Mouse IgG3, λ	Extracellular
CD45	D058-1283	PerCP	BD Biosciences	558411	Mouse IgG1, κ	Extracellular
CD4	L200	APC	BD Biosciences	551980	Mouse IgG1, κ	Extracellular
CD8	SK1	V500	BD Biosciences	561618	Mouse IgG1, κ	Extracellular

[1]Antibody name, clone, color, company, catalogue number, and host of extracellular antibodies used absolute count flow cytometric staining of whole blood.

Supplemental Table S3. Median (95% CI[1]) and p values for statistical analysis of differences in parasitemia between baseline and subsequent time points

	Parasitemia[2]	
**Timepoint**	**Median (95% CI)**	**P value[3]**
Baseline	0.00 (0.00, 0.00)	
Week 2	0.00 (0.00, 0.00)	0.9665
Week 4	0.00 (0.00, 0.00)	0.9665
Week 6	0.00 (0.00, 0.00)	0.9665
Week 8	0.00 (0.00, 0.00)	0.9665
Week 10	0.00 (0.00, 0.00)	0.9665
Week 12A	0.00 (0.00, 0.00)	0.9665
Week 12B	0.00 (0.00, 0.00)	0.9665
Week 12C	0.00 (0.00, 0.00)	0.9665
Week 13A	0.00 (0.00, 0.00)	0.9665
Week 13B	0.1050 (0.00, 0.18)	0.5428
Week 13C	1.435 (0.19, 4.20)	0.6344
Week 14A	25.50 (15.00, 28.00)	**0.0246**
Week 14B	0.00 (0.00, 0.00)	0.9655
Week 14C	0.00 (0.00, 0.00)	0.9655
Week 15A	0.00 (0.00, 0.00)	0.9655
Week 15B	0.00 (0.00, 0.00)	0.9655
Week 15C	0.00 (0.00, 0.00)	0.9655
Week 16A	0.00 (0.00, 0.00)	0.9655
Week 16B	0.00 (0.00, 0.00)	0.9655
Week 16C	0.00 (0.00, 0.00)	0.9655
Week 17A	0.00 (0.00, 0.00)	0.9655
Week 17B	0.00 (0.00, 0.00)	0.9655
Week 17C	0.00 (0.00, 0.00)	0.9655
Week 18A	0.00 (0.00, 0.00)	0.9655
Week 18B	0.00 (0.00, 0.00)	0.9655
Week 18C	0.00 (0.00, 0.00)	0.9655
Week 19A	0.00 (0.00, 0.00)	0.9655
Week 19B	0.00 (0.00, 0.00)	0.9655
Week 19C	0.00 (0.00, 0.00)	0.9655
Week 20	0.00 (0.00, 0.00)	0.9655

[1]CI: Confidence Interval. Data shown is the lower and upper confidence limit of the 95% CI of the median.
[2]Parasitemia is calculated as the % of erythrocytes infected by a parasite among all erythrocytes.
[3]Multiplicity adjusted p values indicating the statistical significance between baseline and subsequent time points. Statistical significance was calculated using a mixed-effects analysis of variance with the Geisser-Greenhouse correction and a Dunnett's multiple comparisons test with individual variances computed for each comparison. P values <0.05 were considered significant and are bolded and highlighted in blue.

Supplemental Table S4. Median (95% CI[1]) and p values for statistical analysis of differences in anemia, plasma viral load and blood CD4+ T cell count between baseline and subsequent time points

	Anemia[2]		Plasma Viral Load[3]		Blood CD4+ T cell count[4]	
**Timepoint**	**Median (95% CI)**	**P value[5]**	**Median (95% CI)**	**P value[5]**	**Median (95% CI)**	**P value[5]**
Baseline	38.50 (37.40, 39.70)		1.00 (1.00, 1.00)		318.10 (168.00, 358.20)	
Week 1			1,803 (1.00, 13.2x10^6)	0.7915		
Week 2	34.70 (32.40, 37.60)	0.0566	9.92x10^6 (3.24x10^6, 15.3x10^6)	0.1386	154.00 (104.00, 311.00)	0.4428
Week 3			11.585x10^6 (5.16x10^6, 24.6x10^6)	0.2315	203.00 (76.00, 205.00)	**0.0190**
Week 4	34.80 (33.00, 37.00)	**0.0383**	5.12x10^6 (2.42x10^6, 30.1x10^6)	0.6647	183.50 (160.00, 213.00)	0.2156
Week 6	36.30 (34.20, 37.90)	0.0957	1.45x10^6 (0.65x10^6, 18.9x10^6)	0.8320	125.80 (103.90, 157.40)	0.1033
Week 8	38.10 (35.50, 43.10)	>0.9999	1.925x10^6 (0.433x10^6, 65.6x10^6	0.9016	140.50 (82.00, 165.00)	**0.0362**
Week 10	37.90 (33.60, 40.60)	0.9537	2,920 (396.00, 0.251x10^6)	0.9203	169.10 (142.30, 182.00)	0.2641
Week 12	37.20 (33.90, 38.50)	0.3894	146 (1.00, 25,600)	0.9261	235.50 (205.00, 333.00)	0.8654
Week 13	36.25 (30.00, 37.30)	0.3373	1,136 (474.00, 36,600.00)	0.8968	332.10 (192.80, 369.40)	0.9773
Week 14	19.80 (13.90, 26.40)	**0.0298**	1,630 (326.00, 9,320.00)	0.6994	385.30 (258.90, 515.10)	0.7720
Week 15	22.80 (19.80, 25.80)	**0.0055**	2,591 (655.00, 35,800.00)	0.8498	241.90 (204.80, 562.30)	>0.9999
Week 16	26.35 (24.00, 30.30)	**0.0270**	809.50 (460.00, 23,700.00)	0.8902	258.2 (225.00, 338.80)	0.9987
Week 17	30.95 (29.50, 35.90)	**0.0370**	869.5 (373.00, 10,700.00)	0.8261	190.70 (127.60, 268.70)	0.4686
Week 18	33.35 (31.20, 39.80)	0.3488	471.00 (1.00, 6,720.00)	0.8629	164.80 (125.00, 184.60)	0.2585
Week 19	35.35 (31.20, 40.50)	0.5131	339.00 (1.00, 9,530.00)	0.8989	163.8 (112.00, 187.90)	0.1075
Week 20	34.65 (32.90, 37.80)	0.1540	1.00 (1.00, 12,500.00)	0.9307	153.70 (148.20, 236.70)	0.2833

[1]CI: Confidence Interval. Data shown is the lower and upper confidence limit of the 95% CI of the median.
[2]Anemia: % hematocrit, defined as the ratio of red blood cells to total blood.
[3]Plasma viral load: Viral RNA copies/ml plasma
[4]Blood CD4+ T cell count: Absolute number of CD4+ T cells per uL of blood.
[5]Multiplicity adjusted p values indicating the statistical significance between baseline and subsequent time points. Statistical significance was calculated using a mixed-effects analysis of variance with the Geisser-Greenhouse correction and a Dunnett's multiple comparisons test with individual variances computed for each comparison. P values <0.05 were considered significant and are bolded and highlighted in blue.

Supplemental Table S5. Median (95% CI[1]) and p values for statistical analysis of differences in CRP, IL-8, IP-10, and MCP-1 between baseline and subsequent time points

	CRP[2]		IL-8[3]		IP-10[4]		MCP-1[5]	
**Timepoint**	**Median (95% CI)**	**P value[6]**	**Median (95% CI)**	**P value[6]**	**Median (95% CI)**	**P value[6]**	**Median (95% CI)**	**P value[6]**
Baseline	0.25 (0.25, 0.25)		2031 (531.10, 4,505.00)		0.4605 (0.4605, 6.83)		76.76 (71.25, 126.50)	
Week 2	0.25 (0.25, 0.25)	0.8854	1,450.00 (900.80, 1,844.00)	0.9102	218.90 (16.86, 391.90)	0.2922	637.90 (166.00, 1,229.00)	0.3096
Week 6	0.25 (0.25, 0.25)	0.8854	2,300.00 (829.00, 4,001.00)	>0.9999	22.31 (0.4605, 39.04)	0.3364	261.40 (71.66, 327.60)	0.3733
Week 8	0.25 (0.25, 0.25)	0.8854	3,795.00 (758.90, 9,141.00)	0.6528	20.63 (8988.00, 48.11)	0.2874	180.10 (120.80, 449.20)	0.5308
Week 10	0.25 (0.25, 0.25)	0.8854	1,803.00 (264.40, 3,943.00)	0.9878	0.4605 (0.4605, 3.67)	0.8854	146.00 (72.27, 152.50)	0.4119
Week 12	0.3950 (0.2500, 0.7100)	0.7672	1,7654 (458.90, 2,771.00)	0.7178	0.4605 (0.4605, 12.10)	0.8854	153.30 (82.70, 200.50)	0.3604
Week 13	1.635 (0.25, 13.71)	0.7672	1,433.00 (615.70, 3,886.00)	0.5186	11.74 (0.4605, 27.80)	0.5553	128.70 (57.13, 178.60)	0.8274
Week 14	30.99 (20.67, 39.70)	**0.0230**	1,613.00 (433.40, 2,119.00)	0.8461	85.98 (20.83, 145.60)	0.2013	471.6 (399.30, 664.40)	**0.0111**
Week 15	0.6950 (0.25, 2.97)	0.6736	1,602.00 (0.61, 2,449.00)	0.5537	14.89 (0.7640, 29.47)	0.5990	200.7 (0.3650, 238.20)	0.7021
Week 16	0.40 (0.25, 15.53)	0.8749	1,791.00 (1,449.00, 3,494.00)	>0.9999	3.151 (0.4605, 10.18)	0.9331	110.6 (90.78, 125.10)	0.5859
Week 20	0.4750 (0.25, 0.77)	0.5581	1,611.00 (373.50, 2,108.00)	0.8891	0.4605 (0.4605, 0.4605)	0.8854	107.2 (82.18, 117.80)	0.6464

[1]CI: Confidence Interval. Data shown is the lower and upper confidence limit of the 95% CI of the median.
[2]CRP: C reactive protein (mg/dL).
[3]IL-8: Interleukin-8 (pg/ml).
[4]IP-10: Interferon gamma-induced protein 10 (pg/ml).
[5]MCP-1: Monocyte chemottractant protein-1 (pg/ml).
[6]Multiplicity adjusted p values indicating the statistical significance between baseline and subsequent time points. Statistical significance was calculated using a mixed-effects analysis of variance with the Geisser-Greenhouse correction and a Dunnett's multiple comparisons test with individual variances computed for each comparison. P values <0.05 were considered significant and are bolded and highlighted in blue.

Supplemental Table S6. Median (95% CI[1]) and p values for statistical analysis of differences in the frequency of total neutrophils and caspase3+ neutrophils between baseline and subsequent time points

	Neutrophil frequency[2]		Caspase3+ neutrophil frequency[3]	
**Timepoint**	**Median (95% CI)**	**P value[4]**	**Median (95% CI)**	**P value[4]**
Baseline	50.05 (49.00, 68.33)		0.8463 (0.5375, 1.033)	
Week 2	54.20 (42.90, 79.30)	>0.9999	0.28 (0.16, 0.60)	0.3485
Week 4	48.55 (37.70, 72.20)	0.9885	1.61 (0.74, 2.53)	0.3850
Week 6	61.30 (44.90, 84.00)	0.6494	0.515 (0.34, 0.96)	0.1532
Week 8	55.15 (39.70, 60.80)	>0.9999	1.575 (0.91, 4.29)	0.5976
Week 10	58.75 (49.20, 76.60)	0.3142	0.56 (0.29, 0.85)	0.6186
Week 12	52.90 (49.20, 76.60)	0.9999	0.9050 (0.70, 1.99)	0.8412
Week 13	51.10 (41.50, 67.20)	>0.9999	0.545 (0.13, 1.21)	0.7244
Week 14	69.40 (62.30, 71.20)	0.1706	0.475 (0.318, 3.00)	0.9996
Week 15	47.35 (33.80, 61.30)	0.8960	0.63 (0.35, 1.35)	0.9999
Week 16	49.05 (44.00, 62.30)	0.9983	1.105 (0.39, 1.89)	0.8565
Week 17	35.35 (29.80, 42.10)	**0.0295**	1.520 (1.39, 2.08)	0.0518
Week 18	50.95 (28.00, 62.30)	0.9057	0.98 (0.60, 2.65)	0.8198
Week 19	42.75 (37.60, 57.30)	0.8481	0.635 (0.35, 0.85)	0.7630
Week 20	56.95 (35.40, 62.00)	>0.9999	0.65 (0.51, 1.71)	>0.9999

[1]CI: Confidence Interval. Data shown is the lower and upper confidence limit of the 95% CI of the median.
[2]Neutrophil frequency: %HLA-DR-CD11b+CD66+CD14+CD49d- Neutrophils of CD45+ cells.
[3]Caspase3+ neutrophil frequency: %Caspase3+ neutrophils among total neutrophils.
[4]Multiplicity adjusted p values indicating the statistical significance between baseline and subsequent time points. Statistical significance was calculated using a mixed-effects analysis of variance with the Geisser-Greenhouse correction and a Dunnett's multiple comparisons test with individual variances computed for each comparison. P values <0.05 were considered significant and are bolded and highlighted in blue.

Supplemental Table S7. Median (95% CI[1]) and p values for statistical analysis of differences in the neutrophil phagocytic score and index between baseline and subsequent time points

	**Neutrophil phagocytic score[2]**		**Neutrophil phagocytic index[3]**	
**Timepoint**	**Median (95% CI)**	**P value[4]**	**Median (95% CI)**	**P value[4]**
Baseline	$0.69x10^6$ ($0.37x10^6$, $1.71x10^6$)		$8.71x10^9$ ($2.29x10^9$, $18.80x10^9$)	
Week 2	$0.59x10^6$ ($0.17x10^6$, $0.74x10^6$)	0.8210	$8.61x10^9$ ($1.88x10^9$, $17.8x10^9$)	>0.9999
Week 4	$0.38x10^6$ ($0.30x10^6$, $1.65x10^6$)	>0.9999	$3.40x10^9$ ($2.31x10^9$, $19.96x10^9$)	>0.9999
Week 6	$1.44x10^6$ ($0.47x10^6$, $1.49x10^6$)	0.6319	$9.90x10^9$ ($5.30x10^9$, $23.50x10^9$)	0.6714
Week 8	$1.02x10^6$ ($0.28x10^6$, $1.49x10^6$)	>0.9999	$9.77x10^6$ ($1.31x10^9$, $17.69x10^9$)	>0.9999
Week 10	$0.102x10^6$ ($0.57x10^6$, $1.97x10^6$)	0.4233	$9.22x10^9$ ($5.89x10^9$, $15.92x10^9$)	>0.9999
Week 12	$0.91x10^6$ ($0.71x10^6$, $1.71x10^6$)	0.3651	$12.20x10^9$ ($8.69x10^9$, $29.55x10^9$)	0.2651
Week 13	$1.01x10^6$ ($0.43x10^6$, $1.36x10^6$)	>0.9999	$11.17x10^9$ ($5.56x10^9$, $12.03x10^9$)	>0.9999
Week 14	$0.93x10^6$ ($0.75x10^6$, $1.55x10^6$	0.9977	$6.73x10^9$ ($4.88x10^9$, $13.74x10^9$)	0.9998
Week 15	$0.70x10^6$ ($0.44x10^6$, $2.22x10^6$)	>0.9999	$8.11x10^9$ ($4.8x10^9$, $21.33x10^9$)	<0.9999
Week 16	$0.71x10^6$ ($0.30x10^6$, $0.98x10^6$)	0.9414	$6.96x10^9$ ($2.54x10^9$, $10.45x10^9$)	0.8230
Week 17	$1.10x10^6$ ($0.30x10^6$, $0.98x10^6$)	0.9977	$10.63x10^9$ ($3.48x10^6$, $17.39x10^9$)	0.9997
Week 18	$0.72x10^6$ ($0.66x10^6$, $1.88x10^6$)	>0.9999	$7.44x10^9$ ($4.97x10^9$, $24.43x10^9$)	>0.9999
Week 19	$0.75x10^6$ ($0.41x10^6$, $1.98x10^6$)	>0.9999	$78.87x10^9$ ($3.52x10^9$, $23.34x10^9$)	>0.9999
Week 20	$0.69x10^6$ ($0.45x10^6$, $1.20x10^6$)	0.9999	$6.39x10^9$ ($4.30x10^9$, $13.07x10^9$)	0.9914

[1]CI: Confidence Interval. Data shown is the lower and upper confidence limit of the 95% CI of the median.
[2]Neutrophil phagocytic score: The number of neutrophils capable of phagocytosis.
[3]Neutrophil phagocytic index: Phagocytic proficiency.
[4]Multiplicity adjusted p values indicating the statistical significance between baseline and subsequent time points. Statistical significance was calculated using a mixed-effects analysis of variance with the Geisser-Greenhouse correction and a Dunnett's multiple comparisons test with individual variances computed for each comparison. P values <0.05 were considered significant and are bolded and highlighted in blue.

Supplemental Table S8. Median (95% CI[1]) and p values for statistical analysis of differences in plasma levels of myeloperoxidase, proteinase 3 and cathepsin G between baseline and subsequent time points

	MPO[2]		PR3[3]		CATG[4]	
**Timepoint**	**Median (95% CI)**	**P value[5]**	**Median (95% CI)**	**P value[5]**	**Median (95% CI)**	**P value[5]**
Baseline	115.40 (98.13, 177.00)		50.85 (22.13, 142.00)		0.6620 (0.5920, 0.7460)	
Week 2	677.60 (518.70, 1,436.00)	0.1369	37.86 (25.65, 79.13)	0.7001	0.6515 (0.5810, 0.9170)	0.9960
Week 6	303.10 (133.60, 707.80)	0.4596	50.43 (10.26, 80,12)	0.8366	0.6375 (0.5430, 0.6780)	0.5768
Week 8	356.10 (152.00, 547.90)	0.2591	39.52 (35.21, 149.50)	>0.9999	0.6375 (0.5880, 1.006)	0.9987
Week 10	111.60 (88.29, 170.80)	0.8849	49.72 (30.68, 169.50)	0.8748	0.6905 (0.5260, 0.7670)	>0.9999
Week 12	84.94 (46.88, 94.47)	0.5401	84.35 (43.92, 225.30)	0.1906	0.6180 (0.5920, 0.8250)	>0.9999
Week 13	142.20 (103.30, 254.80)	0.4516	61.90 (37.31, 175.60)	0.2502	0.5965 (0.5380, 0.7960)	0.9782
Week 14	202.20 (66.96, 315.80)	0.8800	36.73 (24.32, 54.26)	0.7291	0.3395 (0.2410, 0.4340)	**0.0161**
Week 15	132.40 (71.42, 302.30)	0.9917	49.56 (42.87, 58.46)	0.9908	0.52 ().4340, 0.6700)	0.6386
Week 16	212.50 (109.40, 223.00)	0.2493	66.37 (41.38, 120.20)	0.9897	0.6490 (0.5420, 0.9610)	>0.9999
Week 20	90.76 (46.88, 94.79)	0.6271	62.46 (49.92, 99.51)	>0.9999	0.7440 (0.6240, 1.0350)	0.8679

[1]CI: Confidence Interval. Data shown is the lower and upper confidence limit of the 95% CI of the median.
[2]MPO: Myeloperoxidase (pg/ml).
[3]PR3: Proteinase 3 (ng/ml).
[4]CATG: Cathepsin G (ng/ml).
[5]Multiplicity adjusted p values indicating the statistical significance between baseline and subsequent time points. Statistical significance was calculated using a mixed-effects analysis of variance with the Geisser-Greenhouse correction and a Dunnett's multiple comparisons test with individual variances computed for each comparison. P values <0.05 were considered significant and are bolded and highlighted in blue.

Supplemental Table S9. Median (95% CI[1]) and p values for statistical analysis of differences in neutrophil elastase and citrullinated histone 3 between baseline and subsequent time points

	NE[2]		CITH3[3]	
**Timepoint**	**Median (95% CI)**	**P value[4]**	**Median (95% CI)**	**P value[4]**
Baseline	17.00 (10.45, 25.03)		1.34 (0.31, 1.69)	
Week 2	16.07 (12.58, 21.02)	0.9841	0.31 (0.31, 2.19)	0.9778
Week 6	19.15 (10.33, 22.76)	>0.9999	1.241 (0.31, 2.80)	0.9937
Week 8	18.62 (14.94, 25.18)	0.8273	0.31 (0.31, 2.47)	0.9732
Week 10	18.63 (15.55, 20.81)	0.9951	1.825 (0.31, 2.61)	0.4156
Week 12	17.80 (16.37, 19.55)	>0.9999	1.834 (0.31, 2.73)	0.353
Week 13	15.07 (13.40, 25.60)	>0.9999	3.508 (0.31, 5.30)	0.4037
Week 14	33.73 (31.57, 43.55)	**0.0179**	64.97 (10.33, 161.10)	0.4423
Week 15	28.67 (26.39, 29.82)	0.1244	3.334 (0.31, 3.92)	0.5788
Week 16	18.55 (17.89, 24.11)	0.7609	0.31 (0.31, 1.64)	0.8246
Week 20	15.72 (14.03, 21.26)	0.9994	0.886 (0.31, 1.65)	0.9750

[1]CI: Confidence Interval. Data shown is the lower and upper confidence limit of the 95% CI of the median.
[2]NE: Neutrophil elastase (ng/ml).
[3]CITH3: Citrullinated Histone 3 (ng/ml).
[4]Multiplicity adjusted p values indicating the statistical significance between baseline and subsequent time points. Statistical significance was calculated using a mixed-effects analysis of variance with the Geisser-Greenhouse correction and a Dunnett's multiple comparisons test with individual variances computed for each comparison. P values <0.05 were considered significant and are bolded and highlighted in blue.

Supplemental Table S10. Median (95% CI[1]) and p values for statistical analysis of differences in plasma levels of zonulin, intestinal fatty acid binding protein, soluble CD14, and lipopolysaccharide binding protein between baseline and subsequent time points

	Zonulin[2]		I-FABP[3]		sCD14[4]		LBP[5]	
**Timepoint**	**Median (95% CI)**	**P value[6]**	**Median (95% CI)**	**P value[6]**	**Median (95% CI)**	**P value[6]**	**Median (95% CI)**	**P value[6]**
Baseline	21.02 (17.66, 40.72)		5.586 (4.441, 5.652)		790.3 (566.60, 1,062.00)		3,842.00 (2,261.00, 9,747.00)	
Week 2	18.44 (15.35, 22.16)	0.8780	5.198 (2.492, 13.11)	0.9949	756.90 (521.80, 805.80)	0.9481	8,612.00 (2,771.00, 10,392.00)	0.6304
Week 6	15.70 (15.59, 1992.00)	0.9575	4.380 (3.692, 12.10)	0.9986	842.00 (439.20, 1,017.00)	>0.9999	2,225.00 (705.00, 10,556.00)	>0.9999
Week 8	16.36 (15.22, 252.20)	0.9429	4.584 (2.952, 9.796)	>0.9999	1,171.00 (378.00, 1,344.00)	0.7680	2,710.00 (827.30, 12,452.00)	>0.9999
Week 10	19.10 (15.72, 194.80)	0.9468	3.982 (2.292, 5.228)	0.5965	762.20 (567.00, 1,075.00)	>0.9999	4,074.00 (1,261.00, 12,695.00)	>0.9999
Week 12	18.06 (14.61, 137.20)	0.9744	4.574 (3.356, 6.780)	0.9977	1,033.00 (726.30, 1,300.00)	0.2160	1,837.00 (1,735.00, 11,787.00)	>0.9999
Week 13	21.78 (13.35, 104.40)	0.9816	4.920 (2.256, 14.49)	0.9962	932.20 (664.30, 1,135.00)	0.3973	3,031.00 (816.00, 12,805.00)	>0.9999
Week 14	30.07 (10.54, 73.91)	0.9802	14.03 (7.78, 21.40)	0.2016	1,783.00 (847.40, 2,311.00)	0.0975	16,451.00 (13,102.00, 17,233.00)	**0.0367**
Week 15	24.94 (17.32, 38.15)	>0.9999	3.910 (2.172, 19.04)	0.9960	720.70 (592.80, 2,787.00)	0.9392	4,015.00 (523.00, 5,478.00)	0.9560
Week 16	17.32 (14.40, 31.30)	0.9412	3.656 (1.496, 7.440)	0.8655	1,244.00 (750.00, 1,384.00)	0.3051	7,682.00 (1,436.00, 67,390.00)	0.8935
Week 20	17.17 (14.66, 21.81)	0.6847	6.290 (1.764, 8.396)	>0.9999	777.30 (611.40, 1,368.00)	0.9998	1,157.00 (415.00, 4,280.00)	0.7069

[1]CI: Confidence Interval. Data shown is the lower and upper confidence limit of the 95% CI of the median.
[2]Zonulin (ng/ml).
[3]I-FABP: Intestinal fatty acid binding protein (ng/ml).
[4]sCD14: Soluble CD14 (ng/ml).
[5]LBP: Lipopolysaccharide binding protein (ng/ml).
[6]Multiplicity adjusted p values indicating the statistical significance between baseline and subsequent time points. Statistical significance was calculated using a mixed-effects analysis of variance with the Geisser-Greenhouse correction and a Dunnett's multiple comparisons test with individual variances computed for each comparison. P values <0.05 were considered significant and are bolded and highlighted in blue.
